# Interaction of Ions in Organic and Aqueous Media with an Ion-Pair Sensor Equipped with a BODIPY Reporter: An ON1-OFF-ON2-ON3 Fluorescent Assay

**DOI:** 10.3390/ijms24108536

**Published:** 2023-05-10

**Authors:** Marta Zaleskaya-Hernik, Łukasz Dobrzycki, Jan Romański

**Affiliations:** Faculty of Chemistry, University of Warsaw, Pasteura 1, 02-093 Warsaw, Poland

**Keywords:** BODIPY, ion-pair receptors, fluorescent sensors, squaramides, crown ethers

## Abstract

Here, we present a ditopic ion-pair sensor, B1, containing the BODIPY reporter unit in its structure, which is shown to be able—thanks to the presence of two heterogeneous binding domains—to interact with anions in an enhanced manner in the presence of cations. This enables it to interact with salts even in 99% aqueous solutions, making B1 a good candidate in terms of visual salt detection in the aquatic environment. Receptor B1’s ability to extract and release salt was applied in the transport of potassium chloride through a bulk liquid membrane. Working with a concentration of B1 in the organic phase and with the presence of a specific salt in an aqueous solution, an inverted transport experiment was also demonstrated. By varying the type and the amount of the anions added to B1, we were able to develop diverse optical responses, including a unique four-step ON1-OFF-ON2-ON3 output.

## 1. Introduction

**Figure 1:**
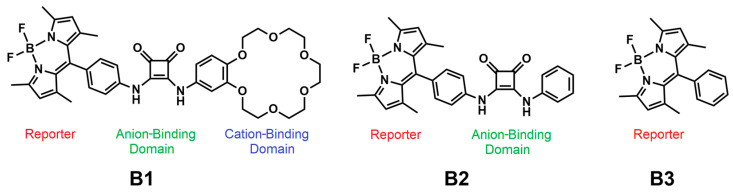
Structures of sensors B1, B2 and B3.

Visual recognition of salts by molecular sensors in an aqueous environment is highly sought after due to the potential for low-cost and easy detection of the desired chemical entities. On the other hand, the challenge of designing switchable fluorescent supramolecular systems that cause an adaptive response is also still of great interest. Boron-dipyrromethene (BODIPY) dyes were first reported in 1968 by Kreuzer and Treibs [1], but their popularity has increased spectacularly over the last two decades. Boron-dipyrromethene derivatives are among the most widely studied fluorescent dyes, due to their high absorption coefficient, high fluorescence quantum yield, and good chemical stability. The derivatives obtained by replacing one or more hydrogen atoms with other functional groups are of great interest as markers, widely used in biological research [2,3,4,5,6]. An additional advantage of BODIPY chromophores is that they can be easily and differently functionalized, and their chemical resistance allows for many post-synthetic modifications of the meso aryl substituent (reduction, oxidation, nucleophilic aromatic substitution) without significant dye decomposition [2,4]. This makes its derivatives an excellent choice as fluorescent building blocks for an interesting substrate in synthesizing new sensor materials. Such single-receptor BODIPY probes have been extensively studied over many years [2,7]. By adding appropriate substituents, water-soluble derivatives or derivatives capable of forming supramolecular gels can be obtained [8,9]. The optical properties of BODIPY dyes in more polar solvents are strongly dependent on the degree of their aggregation and on the mutual orientation of the dye molecules in the aggregates [10,11,12,13,14]. According to Kasha’s theory of molecular excitons, the dipole–dipole interactions between two chromophore units divide the excited state energy in a dimer into two energy levels. The HOMO/LUMO energy gap of the resulting dimer depends on the slip angle and the distance between the centers of the coplanar transition dipoles of the two chromophore units [9,10,11,12]. Thus, the formation of H-type aggregates is possible, due to the flat β-conjugated structure of the boron dipyrromethene core, and strong intermolecular interactions such as π–π stacking and hydrogen bonds are usually observed in the BODIPY aggregation state, leading to a pronounced emission quenching. Moreover, high slip angles θ > 54.7° cause a shift in the blue direction of the main absorption band due to higher excitation energies (“H” after “hypsochromic”) [13,15,16]. On the other hand, as part of J-type aggregation (“J” after E.E. Jelley) [17], the monomer transition dipoles are coplanar with a slip angle θ < 54.7°; they reduce the excitation energy, forming dimers, trimers, or even larger J aggregates [14,18,19]. Wang et al. studied spiro-BODIPY with a diaryl chelate unit that can form J aggregates in an alcohol–water mixture [20]. Some of them showed increased emission efficiency, while structurally altered compounds showed decreased emission efficiency, which suggests that a change in emission intensity is not a reliable indicator of the formation of J aggregates. To explain the factors that govern the formation of BODIPY emission J aggregates, Kim et al. conducted a systematic study of the effect of substitution on meso position. The study showed that the BODIPY packing promotes the formation of J-type aggregates (head to tail) and emits a bathochromic shift compared to their corresponding monomers in solution, while H-type aggregates cause a hypsochromic shift with respect to the monomer in the solution [13,20,21,22]. Some BODIPY derivatives are also used to determine toxic organic compounds—for example, a unit of the BODIPY backbone linked to 1-aza-18-crown-6 capable of binding saxitoxin derivatives [23]. Examples of compounds in which the BODIPY backbone is linked to macrocyclic fragments can be found in the literature. Such compounds are capable of complexing dissimilar metal ions, including potassium [24], iron (III) [25], copper (II) [26,27], mercury [28,29], and other cations [3,4,7]. Moreover, a large group of anion receptors with an integrated boron-dipyrromethene-derivative reporter unit is known [3,6,7,30,31]. However, monotopic receptors (receptors with one type of ion-binding domain) are not always useful in practical applications as they have to compete with the counterion to bind a specific ion. A solution to this problem could be offered by the use of receptors capable of binding both an anion and a cation at the same time, i.e., ion-pair receptors [32,33,34]. To the best of our knowledge, in the literature there is only one reported example of an ion-pair receptor containing the BODIPY derivative [35]. The anion- and cation-binding domains were connected by a boron-dipyrromethene core, which acted as both a chromophore and an electronic mediator between them. The absorption and emission responses were different for the anion and the cation in the inactivity of the other. This behavior is indicative of single responses toward specific ion pairs where the ions do not annihilate each other. Interestingly, the compound was able to distinguish zwitterionic amino acids due to the varied distance between the ammonium group and the carboxylate group. We report here on a new ion-pair sensor, B1 (Figure 1), containing the BODIPY unit in its structure. The anion- and cation-binding domains have been joined in such a way that the cation binding supports the anion-binding strength, leading to cooperative ion-pair binding. For this purpose, the squaramide function (anion-binding site) obtainable by sequential amidation of dialkyl squarate [36,37] was decorated successively with the BODIPY unit (reporter) and benzo crown ether (cation-binding site). To test the individual importance of each structural part of B1, anion sensor B2, lacking the cation-binding domain, and compound B3, lacking ion-binding domains, were also tested.

## 2. Results and Discussion

### 2.1. Receptor Design and Synthesis

**Scheme 1:**
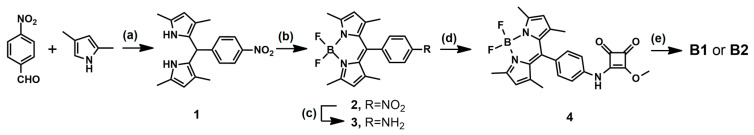
Synthesis of sensors B1 and B2. Reagents and conditions: (a) trifluoroacetic acid, dry THF, 24 h, argon atmosphere, r.t.; (b) DDQ, BF_3_·OEt_2_, triethylamine, dry THF, 24 h, argon atmosphere, 0 °C to r.t., 69% yield; (c) SnCl_2_ × 2H_2_O, ethanol, 70 °C, 2 h, 94% yield; (d) dimethyl squarate, methanol, r.t., 24 h, 63% yield; (e) 4-aminobenzo-18-crown-6 or aniline, triethylamine, methanol, r.t., 24 h, 70% and 59% for B1 and B2, respectively.

The synthesis of receptors B1 and B2 is outlined in Scheme 1. Compound **1** was prepared in accordance with the procedure described in the literature [38]. Briefly, the *p*-nitrobenzoaldehyde was coupled with 2,4-dimethylpyrrole in the presence of trifluoroacetic acid. After the oxidation of 1 with 2,3-dichloro-5,6-dicyano-1,4-benzoquinone was reacted with BF_3_·OEt_2_ in the presence of triethylamine, yielding compound **2**. The nitro group in compound **2** was reduced with SnCl_2_ × 2H_2_O in ethanol giving amine 3, which was reacted with dimethyl squarate affording monoester 4 with 63% yield. A subsequent reaction of monoester 4 with 4-aminobenzo-18-crown-6 or with aniline gave sensors B1 or B2 with 70% and 59% yield, respectively. 

### 2.2. Binding Studies in Organic Solvents

**Figure 2:**
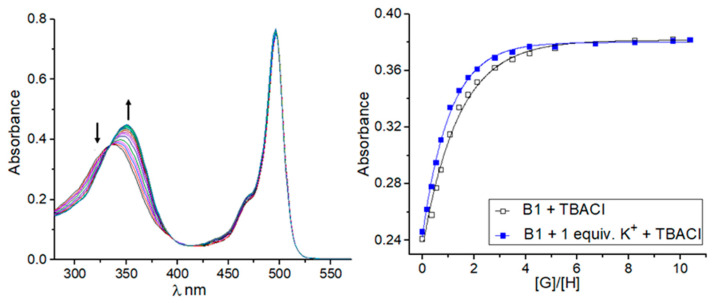
Representative binding behavior of receptor B1 upon gradual addition of TBACl: UV–Vis titration spectra and corresponding experimental (points) and theoretical (lines) binding isotherms (λ = 360 nm) for TBACl alone and with the presence of 1 equivalent of KPF_6_.

In order to assess the ion-binding properties of the receptors obtained, UV–Vis spectroscopic titration experiments in acetonitrile were conducted. At first, the affinity of sensors B1 and B2 toward chloride anions and sodium and potassium chlorides was investigated. In the case of the interaction of the sensors with chloride anions, tetrabutylammonium salt was used (TBACl). Due to the low solubility of sodium and potassium salts in acetonitrile, the interaction of the sensors with these salts was performed by generating the salts in situ. This involved titration experiments with anions being carried out in the presence of one equivalent of the corresponding cations (sodium perchlorate or potassium hexafluorophosphate were added as a source of sodium or potassium cations, respectively). When solutions of the receptors were titrated with TBACl in the presence and absence of the cation, a bathochromic shift of the absorption maximum centered at 340 nm was observed (Figure 2). By fitting the UV–Vis spectroscopic titration curves to a standard 1:1 binding profile, association constants (K_a_) corresponding to the interactions between sensors B1 or B2 and chlorides were determined. The values of stability constants were found to be 1.60 × 10^5^ M^−1^ and 2.91 × 10^5^ M^−1^ for complexes of B1 and B2 with chloride anions, respectively. The lower stability constant value for B1 can be attributed to the presence of electron-donating alkoxylate substituents (crown ether) in the sensor B1 structure diminishing the ability of the squaramide unit to form hydrogen bonds. However, in the presence of sodium or potassium cations, an enhancement in chloride anion binding was observed, which in the case of B1 produced apparent stability constants of 3.06 × 10^5^ M^−1^ and 3.40 × 10^5^ M^−1^ for complexes with in situ generated sodium and potassium chlorides, respectively. On the other hand, the monotopic sensor B2 was able to recognize chloride anions less effectively in the presence of cations with apparent association constants equal to K_NaCl_ = 1.45 × 10^5^ M^−1^ and K_KCl_ = 1.70 × 10^5^ M^−1^. These preliminary results confirm the validity of the construction of the ditopic receptor for the effective binding of the ion pairs and the selectivity of sensor B1toward potassium salts. This was further confirmed by titration experiments of B1 with sodium perchlorate and potassium hexafluorophosphate, which show the preference of B1 for potassium cations (K_K+_ = 6.22 × 10^4^ M^−1^ vs. K_Na+_ = 3.50 × 10^4^ M^−1^).

The addition of tetrabutylammonium perchlorate or tetrabutylammonium hexafluorophosphate to the solution of B1 in acetonitrile does not affect its spectral properties, thus confirming no interactions of B1 with these ions in such conditions. Considering the above, further titration experiments were extended to the selected anions and sensor B1 in the absence and presence of potassium cations (Table 1). The investigated anions were bound to receptor B1 moderately, in the order Br^−^ < NO_3_^−^ < NO_2_^−^ < Cl^−^. In the presence of a potassium cation, each anion was more strongly bound with B1, and the highest enhancement factor (defined as the ratio of the value of the stability constant calculated by titration experiments carried out in the presence of potassium cations to the value of the stability constant calculated for complexes of B1 with anions) was found for bromide and chloride anions (see supporting information, Figures S7–S21). A divalent sulfate anion was also tested; however, the titration data collected did not fit any appropriate binding model, suggesting more complex equilibria in solution in this case [39,40,41].

In order to better establish the binding mechanism of B1 with ions, titration experiments under ^1^H NMR spectroscopic control were performed. We found that in the concentration range required by NMR measurements, B1 creates intermolecular interactions in acetonitrile. This was manifested by upfield shifts of the signals corresponding to the squaramide protons upon dilution and a slightly lower-than-calculated diffusion coefficient determined for B1 in acetonitrile [42]. For this reason, ^1^H NMR titration experiments of B1 with anions were performed in the more competitive solvent DMSO-d_6_. We selected Cl^−^ and NO_3_^−^ as representatives of anions forming the weakest and strongest complexes with B1. In the case of titration of B1 with nitrates, the change in the chemical shifts of the signals corresponding to squaramide signals were so small that it was not possible to calculate the stability constant of the complex formed. In contrast, upon the addition of chloride anions to the solution of B1 in DMSO-d_6_, the NH signals were shifted significantly, with Δ δ over 1.20 ppm. Importantly, when the receptor was pre-treated with one equivalent of potassium cations, the signals corresponding to the crown ether unit as well as to both squaramide NHs were moved downfield, evidencing both the interaction of the potassium cation with the crown ether and the cation-induced increase in the acidity of the squaramide protons at the same time. On the basis of the shifts of the signals corresponding to squaramide and aromatic protons, the stability constants were determined to be 565 and 722 M^−1^ in the absence and presence of one equivalent potassium cation, respectively.

### 2.3. Crystallization and Single-Crystal X-ray Diffraction

**Figure 3:**
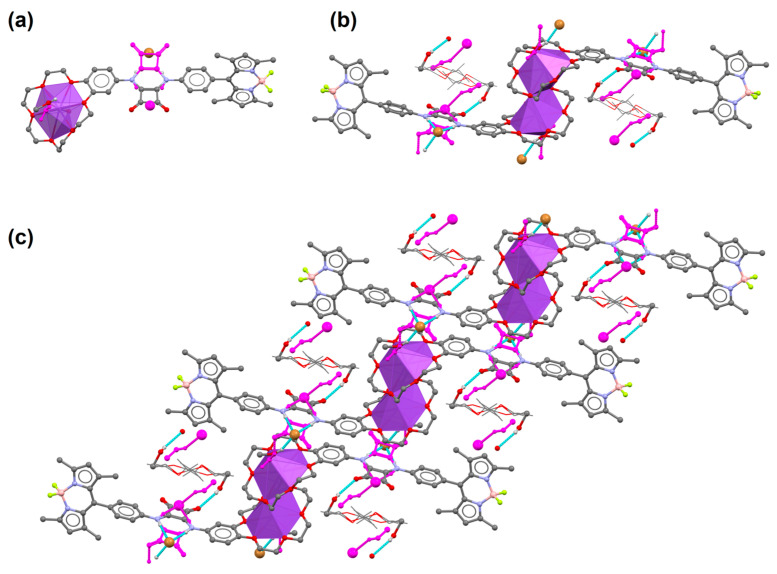
(**a**) Basic subunit of B1 coordinating NaBr/KBr salt, (**b**) dimeric units of B1 observed in the crystal lattice which form (**c**) 1D coordinating polymers. Magenta color denotes alternative positions of the disordered species associated with K^+^ ion presence. Most hydrogen atoms are omitted for clarity. Disordered diethyl ether molecules are presented as a wireframe model.

Direct evidence that B1 forms adducts with an ion pair in the solid state, confirming the binding capabilities of the receptor, was obtained from a single-crystal X-ray diffraction experiment. CCDC 2226366 contains the supplementary crystallographic data for this paper. These data can be obtained free of charge from The Cambridge Crystallographic Data Centre via www.ccdc.cam.ac.uk/structures (accessed on 29 April 2023). Receptor B1 crystallizes with Br- ions in the form of a mixture of Na^+^/K^+^ salt with a significant excess of sodium species in the structure. Na^+^ cations in the intended crystallization of B1 with KBr come from the sodium lab glassware used during experimental procedures. The NaBr/KBr ratio yields 0.915(1):0.085(1). Both cations share the same positions sitting in the crown ether moiety. The positional disorder of cations is associated with the alternative orientation of the squaramide unit binding Br- anions, which are visualized in Figure 3a. Cations are additionally coordinated from one side of the crown ether by water (K^+^) or MeOH (Na^+^). From the other side, the cations are stabilized by one of the ethoxy O atoms of the neighboring crown ether, giving centrosymmetric dimers Figure 3b. Such dimers are extended in [110] direction via hydrogen bonds between MeOH/H_2_O cation coordinating species and Br^−^ anions, which are visible in Figure 3c. The structure contains additional MeOH and diethyl ether molecules (see supporting information, Figures S27 and S28 and Table S2).

### 2.4. Studies in the Aqueous Environment

**Figure 4:**
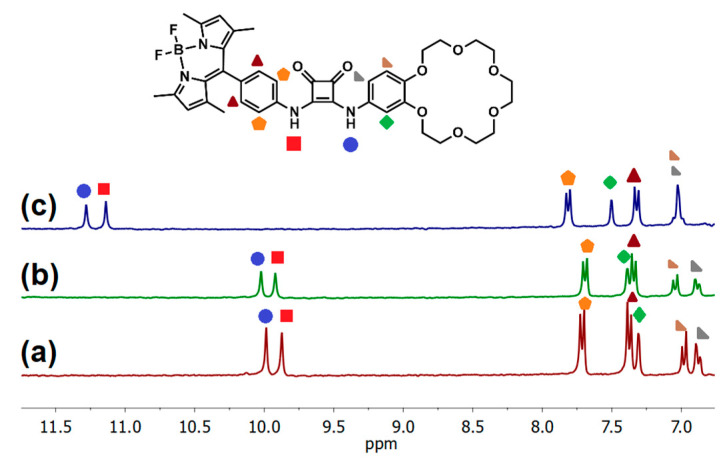
(**a**) Partial ^1^H NMR spectra of 3 mM receptor B1 recorded in DMSO-d_6_; (**b**) after the addition of 10% aqueous 0.5 M KNO_3_; and (**c**) after the addition of 10% aqueous 0.5 M KCl.

The next step was to verify whether B1 can interact with salt in an aqueous environment. The addition of 10% (*v*/*v*) of water to the 3.0 mM solution of B1 in DMSO caused the precipitation of the receptor. Interestingly, after adding 10% (*v*/*v*) 0.5 M potassium nitrate or potassium chloride solution, the solution remained homogeneous, allowing further analysis. ^1^H NMR analysis clearly shows that in an aqueous environment B1 can interact with potassium chloride. This was manifested by the downfield shift of the signals corresponding to the squaramide protons by approx. 1.2 pm after the addition of aq. KCl, relative to the addition of KNO_3_, which was shown to interact with B1 only slightly in an H_2_O/DMSO mixture (Figure 4).

**Figure 5:**
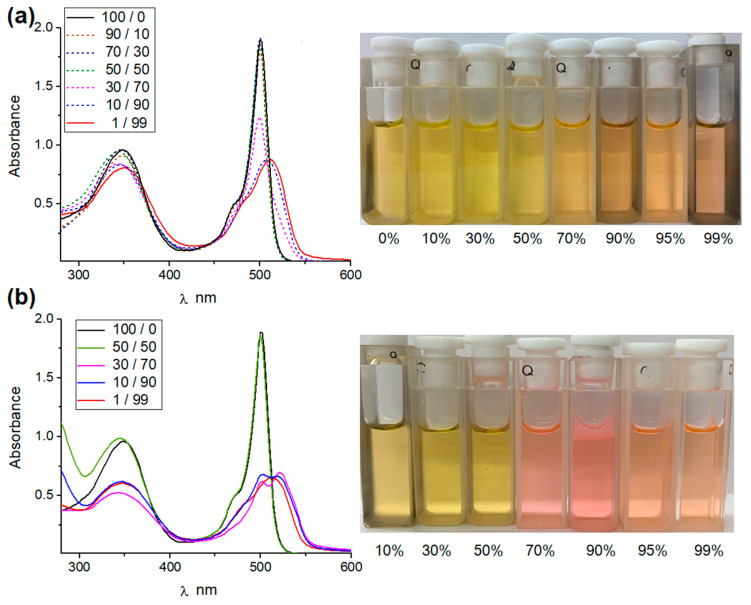
(**a**) Absorption spectra of 2.0 × 10^−5^ B1 in DMSO/water; (**b**) B1 in DMSO/0.5 M aq. KCl.

Carrying out the titration experiment in the modified system, namely in the presence of a 10% aqueous 0.5 M KNO_3_ in the DMSO-d_6_ solution, also made it possible to determine the apparent stability constant for complexes of B1 with Cl^−^ (K_TBACl_ = 61.5 M^−1^). By reducing the concentration of B1 by two orders of magnitude, we were able to dissolve the receptor in up to 99% of water and investigate its behavior under UV–Vis control (Figure 5). Upon increased water fraction in the solution of B1, the peak derived from the BODIPY unit shifted into red waves from 504 nm in DMSO to 517 nm in a 1/99 DMSO/water mixture. These observations suggest that J aggregates start to form from a fraction of water of 70%, which is justified, as B1 has poor water solubility, and more aggregates are formed at a constantly increasing fraction of water value (Figure 5a) [13,20]. A similar phenomenon occurs in the case of the addition of 0.5 M aqueous KCl solution to the solution of B1 in DMSO. From the fraction of a 70% aq. KCl in DMSO, a well-defined second absorption maximum from the BODIPY unit appears toward the red waves (Figure 5b).

**Figure 6:**
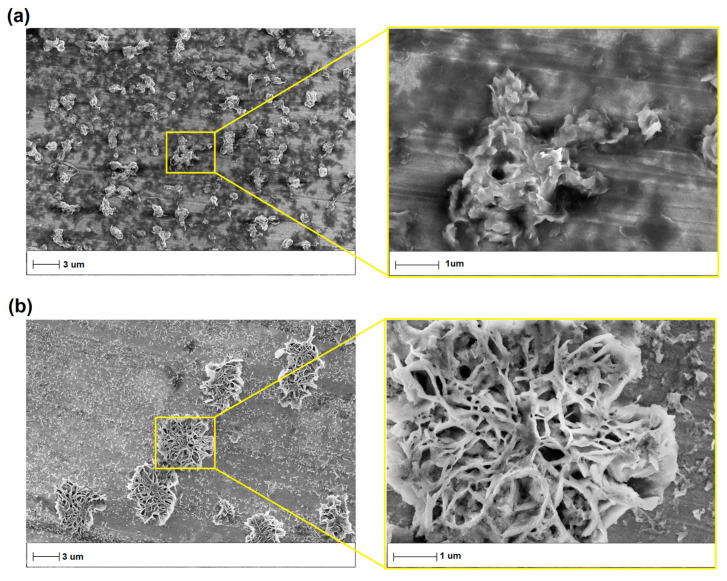
SEM images of (**a**) B1 in a 90% (*v*/*v*) water/DMSO mixture; (**b**) B1 in a 90% (*v*/*v*) 0.5 M aq. KCl/DMSO mixture.

Interestingly, the color change from yellow to pink solution was noticeable in daylight, which was not observed in the experiment carried out with B1 in water/DMSO mixtures. This behavior may indicate the prospect of the ability of such systems to visually detect salts in an aqueous environment. The continuous redshift of the above absorptions at an increased level of aqueous solution at a constant concentration of the B1 receptor suggests an improvement in the packing order of BODIPY molecules in aggregates. This was confirmed by dynamic light scattering (DLS) experiments (see supporting information, Figure S41). For sensor B1 in a 90% water in DMSO mixture, the hydrodynamic radius value was determined to be approx. 40 nm, while in the case of a 90% aq. KCl/DMSO mixture, this value increased to 300 nm. This may suggest possible additional interactions of receptor hydrogen bonding and KCl (see supporting information, Figure S42), which, in combination with J aggregation derived by BODIPY molecules, facilitate the formation of even larger J aggregates. Scanning electron microscopy (SEM) images of the dried aggregates formed from B1 and B1 × KCl in a 90% (*v*/*v*) water/DMSO mixture confirm this assumption, although these results should be treated with caution, as the morphology may have changed during drying (Figure 6). On both occasions, the formation of self-assemblies was observed, consisting of aggregated particles of approx. 50–70 nm for B1, while in the case of B1 × KCl, the aggregates exhibited a more condensed morphology, as well as the formation of crystalline structures within the film.

### 2.5. Extraction Studies

Further efforts were made to apply B1 as an extractant of potassium salts (KCl, KBr, KNO_3_, K_2_SO_4_) in liquid–liquid extraction experiments using aqueous solutions of selected salts and a chloroformic solution of receptor B1. Direct comparison of B1 with B2 failed due to the low solubility of the latter compound in chloroform. At first, B1 was investigated qualitatively under ^1^H NMR control. It was found that, after extraction of the chloroformic solution of B1 (3.0 mM) with aqueous solutions of salts (20 mM), the signals corresponding to the squaramide protons were moved downfield, evidencing the participation of this function in anion binding. Perturbation in the signals corresponding to the crown ether unit was also detected, confirming that B1 is able to extract cations and anions simultaneously. Interestingly, the most pronounced changes in the ^1^H NMR spectrum after extraction was noted for potassium sulfate extraction (see supporting information, Figures S33, S44 and S45). Additionally, back extractions with deionized water were performed for each case, after which the organic layer was examined. Squaramide signals returned to almost the same position as in the initial case, namely in the spectrum registered for the solution of B1 in wet chloroform. This experiment suggests that B1 is able to extract and release salts and may be used as a promising transporter. In order to estimate the extraction efficiency, a series of extraction experiments were performed under ion chromatography (IC) control. This time, a 20 mM chloroformic solution of B1 was used, and the aqueous solutions of potassium salts were decreased to 5 mM to enable tracking of the loss of salts by IC. The drop in salt concentration after extraction was found to be 26%, 34%, 27%, and 29% for potassium chloride, bromide, nitrate, and sulfate, respectively.

### 2.6. Transport Experiments

**Figure 7:**
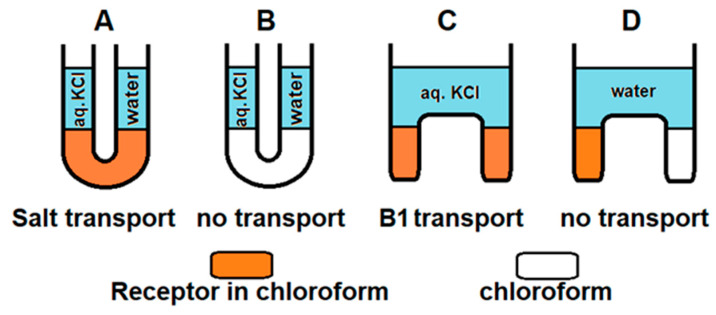
Schematic illustration of transport experiments. (**A**) KCl transport across membrane consisting of B1 in chloroform. (**B**) KCl transport across membrane consisting of chloroform. (**C**) B1 transport across membrane consisting of 0.5 M KCl. (**D**) B1 transport across membrane consisting of water.

Considering the ability of B1 to extract salts and release them, we conducted transport experiments using a bulk liquid membrane (U-tube). A 15 mM receptor B1 solution in chloroform was used as a membrane, and a 200 mM aqueous solution of potassium chloride was placed in the source phase. The salt concentration in the receiving phase was monitored by conductometry. After 9 days, B1 transported potassium chloride across the membrane with 56% yield. Interestingly, due to the sufficient solubility of B1 in a concentrated solution of aq. KCl, it was possible to construct inverted transport experiments. For this purpose, a 15 mM solution of B1 in CDCl_3_ was used as the source phase, CDCl_3_ was used as the receiving phase, and these two phases were separated with a membrane consisting of 0.5 M KCl solution in water. After 7 days, the receiving phase became colored and the ^1^H NMR spectrum recorded for the solution in the receiving phase showed specific signals corresponding to B1 (see supporting information, Figure S43). The signals corresponding to the squaramide protons of B1 were shifted downfield, indicating the fraction of the receptor in the form of B1⸦KCl complex in the receiving phase. Control experiments using pure water as a membrane demonstrated the inability to transport B1 effectively through water without ions. This shows that by varying receptor concentration in the organic phase and KCl concentration in the aqueous layer, B1 may be an effective salt transporter and, vice versa, KCl may transport B1 across the aqueous layer (Figure 7).

### 2.7. Fluorescent Studies

**Figure 8:**
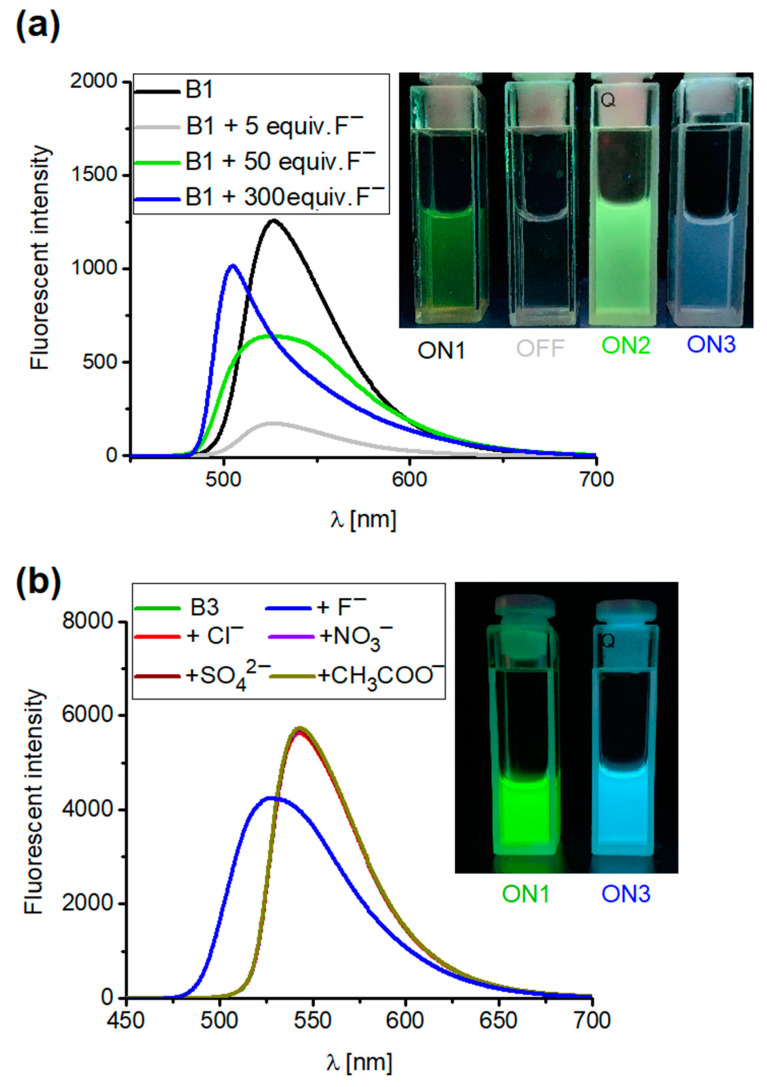
Emission spectra of (**a**) receptor B1 upon addition of 5, 50, and 300 equiv. of TBAF and (**b**) B3 (2.0 × 10^−5^ M) upon addition of 100 equiv. of salt TBA (excitation at 496 nm) in CH_3_CN.

Given the presence of the BODIPY unit in receptor B1’s structure, in further studies we decided to investigate the fluorimetric response of this sensor toward anions. The emission spectrum of B1 is characterized by a broad band centered at 526 nm (λ_exc._ = 496 nm) in acetonitrile at room temperature. When the anions were added as TBA salts, a different fluorimetric response of B1 was observed (see supporting information, Figure S46). Upon the addition of 100 equivalents of chloride, bromide, nitrate, and nitrite anions (added as TBA salts) to the solution of B1 in acetonitrile, the fluorescent intensity decreased, with the highest drop in intensity seen after the addition of chloride anions. This correlates well with the most stable complexes of B1 with chloride salts formed within this series. Interestingly, when adding 100 equiv. sulfate or carboxylate salts or 5 equiv. of fluoride anions to the B1 solution in acetonitrile, the fluorescence was quenched, which could be detected with the naked eye using a UV–Vis lamp (see supporting information, Figures S46 and S48). The process of quenching the fluorescence emission band was attributed to an electron-transfer (eT) process recently reported for the interaction of basic anions with the fluorescent urea- or squaramide-based receptors [43]. The nature of the electron-transfer (eT) process was also established for the interaction of fluorescent squaramides with sulfate salts [41]. Such a mechanism is known to be related to the separation of electrical charges and consists of the reorganization of the solvent molecules bound to the receptor, where the creation of conditions immobilizing the solvent molecules causes the fluorescence to revive. To ensure the same process for B1, after the addition of anions the quenched samples were cooled down to approx. 0 °C. As expected, the quenched emission was raised back up at the reduced temperature, which proves the assumed eT process (see supporting information, Figures S49 and S50) [44]. Due to the adaptive response, there is still significant interest in designing switchable fluorescent supramolecular systems, and ON-OFF, OFF-ON or ON1-OFF-ON1 or OFF-ON-OFF responses are being developed. ON1-OFF-ON2 outputs are also available, but only a few circuits have been described in the literature [45,46,47,48,49,50,51,52,53,54,55]. To fill this niche, we decided to gain deeper insight into the nature of the interaction of the fluoride anion with B1 and verify whether a new unique ON1-OFF-ON2-ON3 output can be developed. To the best of our knowledge, such systems have never previously been reported. Thus, we carried out fluorescence titration experiments in acetonitrile and found that four outputs can be clearly distinguished. The corresponding intermediates were recognized by ^1^H, ^19^F, and ^11^B NMR and UV–Vis spectroscopy (see Supporting Information Figures S38–S40, S53 and S54). The initially emissive B1 (ON1) was quenched upon the addition of 5 equivalents of fluoride anions (OFF output) due to an eT process (Figure 8a). This process was proved by restoring fluorescence at decreased temperature after quenching (see supporting information, Figure S50). Furthermore, after the addition of the first portion of fluoride anions, the signals corresponding to the squaramide protons in the ^1^H NMR spectrum were shifted downfield, indicating complex formation (see supporting information, Figure S38). The addition of more equivalents of F^-^ to the solution of B1 in CD_3_CN caused the disappearance of the signals corresponding to the squaramide protons in the ^1^H NMR spectrum as a consequence of the deprotonation event, according to the equilibrium [B1H···F]^−^ + F^−^ ⇆ [B1]^−^ + HF_2_^−^. This produced anionic species with green fluorescence with an extended band centered at 520 nm (ON2). Finally, the addition of excess fluoride anions caused a change to blue fluorescence with a band centered at 502 nm. We attributed the ON3 output to the disruption of the stable difluoroboron bridges, due to nucleophilic displacement and the breaking of the BN bond, when larger quantities of fluoride ions were added [56]. To verify this, we monitored the changes in the ^11^B and ^19^F spectra after the addition of the excess fluoride anions to the solution of B1. Indeed, the characteristic triplet at 0.75 ppm disappeared in the ^11^B spectrum for B1 (2 mM) in CD_3_CN. In the ^19^F spectrum, the quartet signal at −145.4 ppm disappeared, while the two new singlet peaks increased at −115.5 ppm and −153.5 ppm, where the signal at −153.5 corresponds to HF_2_^-^ formed and the signal at −115.5 corresponds to fluoride from excess added TBAF salt [31,57] (see supporting information, Figures S39 and S40). The disruption of the stable difluoroboron bridge was also proved by UV–Vis spectroscopy, with the band corresponding to the BODIPY unit vanishing after the addition of excess fluoride anions to the solution of B1 (see supporting information Figures S53 and S54). Analogous changes were observed for the reference compound, B3, lacking ion-binding domains (see supporting information, Figures S34 and S35).

**Figure 9:**
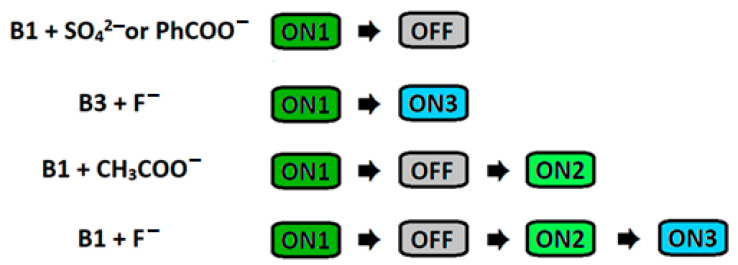
Schematic representation of different fluorescent outputs obtained by using receptors and various anions.

Due to the absence of squaramide function in the structure of B3, the OFF and ON2 outputs were omitted, and only the ON1-ON3 assay could be developed as a result of the disruption of the stable difluoroboron bridge (see supporting information, Figures S51 and S52). Because B3 does not possess an anion-binding domain, other anions do not alter their spectral properties, thus demonstrating the selectivity of B3 toward fluoride anions (Figure 8b). Interestingly, the ON1-OFF output established for the interaction of B1 with acetate anions could be extended to the ON2 state as a result of the deprotonation of B1 in such conditions. This was not the case with benzoate anions, in that the addition of an excess of this salt to the solution of B1 maintained the quenching (OFF), thus allowing these two carboxylates to be discriminated (see supporting information, Figures S32, S36, S37 and S47). Thus, by playing with the sensors and anions, different optical responses could be developed, including the unique ON1-OFF-ON2-ON3 system, which to the best of our knowledge has never previously been reported (Figure 9).

## 3. Materials and Methods

### 3.1. Materials and Reagents

All reagents and chemicals were of reagent grade quality and purchased commercially. ^1^H, ^19^F, ^11^B, and ^13^C NMR spectra were recorded on a Bruker 300 MHz spectrometer. ^1^H and ^13^C NMR chemical shifts δ are reported in ppm referenced to residual solvent signal (see supporting information, Figures S1–S6 and S29–S31). References standards for ^19^F and ^11^B were used, namely CFCl_3_ and BF_3_ × OEt_2_, respectively. Mass spectra were measured on a Quattro LC Micromass or a Shimadzu LCMS-IT-TOF unit. Unless specifically indicated, all other chemicals and reagents used in this study were purchased from commercial sources and used as received. If necessary, purification of products was performed using column chromatography on silica gel (Merck Kieselgel 60, 230–400 mesh) with mixtures of chloroform/methanol. Thin-layer chromatography (TLC) was performed on silica gel plates (Merck Kieselgel 60 F254).

### 3.2. UV–Vis Experiments

UV–Vis titration experiments were performed in CH_3_CN solution at 298K using a Hitachi U-2910 spectrophotometer. To a 10 mm cuvette was added 2.5 mL of freshly prepared (B1 − c = 1.0 × 10^−5^ M; B2 − c = 1.1 × 10^−5^ M) solution of the studied receptor and, in the case of ion-pair-binding studies, 1 mol equivalent of cation (KPF_6_ or NaClO_4_) was added before titrations. Small aliquots of ca. 1.0 × 10^−3^ M TBAX solution containing the receptor at the same concentration as in the cuvette were then added, and a spectrum was acquired after each addition. The resulting titration data were analyzed using the BindFit (v0.5) package, available online at http://supramolecular.org (accessed on 10 November 2022). Each titration was carried out in duplicate. Reported values were calculated as a weighted arithmetic mean, where the weights were the errors obtained for each value separately. The given uncertainty of the association constants was the largest of the variance (external or internal).

### 3.3. ^1^H NMR Experiments

The ^1^H NMR titration was conducted at 298 K in DMSO-d_6_. In each case, a 500 μL of freshly prepared 1.9 mM solution of B1 was added to a 5 mm NMR tube. In the case of ion-pair titration, receptor B1 was firstly pre-treated with one equivalent of KPF_6_. Then small aliquots of a solution of TBAX, containing the receptor at a constant concentration, were added, and a spectrum was acquired after each addition. The resulting titration data were analyzed using the BindFit (v0.5) package, available online at http://supramolecular.org (accessed on 10 November 2022). See supporting information, Figures S22–S26.

### 3.4. Liquid–Liquid Extraction (LLE)

A solution of receptor B1 in chloroform (2 mL, 20 mM) was intensively shaken with the aqueous mixture (no pH adjustment, pH depending on the salts used; above pH 8 there is no phase separation, probably due to the receptor deprotonation). This eliminates the direct use of basic salts such as carboxylates, hydrogen phosphates, or phosphates. The receptor solution was shaken vigorously with the corresponding 5 mM saline solution (2 mL) for 5 min. Then 1 mL of the aqueous phase was taken and tenfold diluted. The concentration of chloride, bromide, nitrite, nitrate, dihydrogenphosphate, and sulfate anions in the aqueous phase was determined by high performance ion chromatography (HPIC) using a 930 Compact apparatus (see supporting information, Table S1).

### 3.5. Synthetic Details

**Preparation of compound 2.** Under an argon atmosphere, p-nitrobenzaldehyde (0.9 g, 6.0 mmol) was added to a solution of 2,4-dimethylpyrrole (1.25 g, 13.2 mmol) in dry THF (200 mL), and the mixture was stirred for 1 h at room temperature. Then, 0.2 mL of trifluoroacetic acid was dropped into the reaction mixture, and the stirring continued in the dark for 24 h under an argon atmosphere at room temperature. After the complete consumption of p-nitrobenzaldehyde (monitored by TLC), the crude product of compound **1** was used in the next step without further purification. To the solution of compound **1** (1.9 g, 5.9 mmol) in dry THF (200 mL) was added, drop by drop, 2,3-dichloro-5,6-dicyano-1,4-benzoquinone (1.36 g, 6.0 mmol) in dry THF (200 mL). After being stirred for 5 h, triethylamine (36 mL) and BF_3_·OEt_2_ (36 mL) were added slowly to the reaction mixture in an ice bath. After the mixture was further stirred for 12 h, it was filtered. The solid obtained was washed with THF, acetone, and dichloromethane. The combined organic layers were concentrated under reduced pressure. The product was purified by column chromatography (2% methanol in dichloromethane) to give compound **2** as a red solid (1.53 g, 4.15 mmol, 69%). HRMS (ESI): calcd for C_19_H_18_BF_2_N_3_O_2_Na [M + Na]^+^ 392.1358; found: 392.1363. ^1^H NMR (300 MHz, DMSO-d_6_, δ ppm): 8.52–8.35 (d, J 9 Hz, 2H), 7.85–7.64 (d, J 9 Hz, 2H), 6.23 (s, 2H), 2.56–2.40 (s, 6H), 1.35 (s, 6H). ^13^C NMR (75 MHz, DMSO-d_6_, δ ppm): 156.2, 148.6, 143.1, 141.2, 139.7, 130.5, 130.4, 124.8, 122.2, 14.7, 14.7.

**Preparation of compound 3.** To a solution of compound **2** (0.62 g, 1.7 mmol) in ethanol (10 mL) was added SnCl_2_ × 2H_2_O (1.9 g, 8.4 mmol). The reaction mixture was flushed with argon and heated to 70 °C for 1 h, then allowed to cool down, and then poured into ice. The pH was made basic to 10 by the addition of 5% aqueous NaOH, and the resulting basic mixture was kept for one hour under stirring. The aqueous mixture was extracted four times with ethyl acetate; the organic phases were collected, washed with brine, and dried over anhydrous MgSO_4_. The solvent was then removed under reduced pressure to give compound **3** as a maroon solid (0.54 g, 1.6 mmol, 94%). HRMS (ESI): calcd for C_19_H_21_BF_2_N_3_ [M + H]^+^ 340.1797; found: 340.1790. ^1^H NMR (300 MHz, DMSO-d_6_, δ ppm): 6.88–6.96 (d, J 9 Hz, 2H), 6.65–6.73 (d, J 9 Hz, 2H), 6.15 (s, 2H), 5.40 (s, 2H), 2.43 (s, 6H), 1.48 (s, 6H). ^13^C NMR (75 MHz, DMSO-d_6_, δ ppm): 154.4, 150.0, 144.3, 143.1, 131.8, 128.7, 121.4, 120.8, 114.5, 14.7, 14.6.

**Preparation of compound 4.** To a solution of compound **3** (0.54, 1.6 mmol) was added dimethyl squarate (0.23 g, 1.6 mmol) in methanol (20 mL). After being stirred for 24 h at room temperature, the reaction was concentrated and purified by silica gel column chromatography (2% methanol in dichloromethane) to give compound **4** as an orange-red solid (0.45 g, 1.0 mmol, 63%). HRMS (ESI): calcd for C_24_H_21_BF_2_N_3_O_3_ [M − H]^−^ 448.1649; found: 448.1630. ^1^H NMR (300 MHz, DMSO-d_6_, δ ppm): 10.94 (s, 1H), 7.61 (d, J 8 Hz, 2H), 7.38 (d, J 8 Hz, 2H), 6.19 (s, 2H), 4.41 (s, 3H), 2.46 (s, 6H), 1.41 (s, 6H). ^13^C NMR (75 MHz, DMSO-d_6_, δ ppm): 188.2, 184.6, 179.7, 169.6, 155.3, 143.2, 142.1, 139.5, 131.3, 129.7, 129.3, 121.8, 120.2, 61.2, 14.7.

**Preparation of receptor B1.** 4-Aminobenzo-18-crown-6 was synthesized according to the literature protocol [41]. Under an argon atmosphere, 4-aminobenzo-18-crown-6 (0.33 g, 1.0 mmol) and compound **4** (0.45 g, 1.0 mmol) were dissolved in methanol (20 mL), and the mixture was stirred for 24 h at room temperature, in the presence of triethylamine (0.42 mL, 3.0 mmol). Then the reaction was concentrated and purified by silica gel column chromatography (10% methanol in dichloromethane) to give receptor B1 as an orange solid (0.52 g, 0.7 mmol, 70%). HRMS (ESI): calcd for C_39_H_43_BF_2_N_4_O_8_Na [M + Na]^+^ 767.3047; found: 767.3058. ^1^H NMR (300 MHz, DMSO-d_6_, δ ppm): 10.92 (s, 1H), 10.76 (s, 1H), 7.82 (d, J 9 Hz, 2H), 7.45–7.30 (m, 3H), 6.97 (m, 2H), 6.20 (s, 2H), 4.20–4.05 (d, 4H), 3.85–3.70 (m, 4H), 3.70–3.50 (m, 12H), 2.46 (s, 6H), 1.46 (s, 6H). ^13^C NMR (75 MHz, DMSO-d_6_, δ ppm): 193.3, 182.5, 181.2, 175.6, 174.3, 166.5, 165.7, 165.5, 155.2, 149.2, 145.0, 143.9, 143.2, 140.5, 131.5, 129.5, 121.8, 119.0, 114.6, 111.0, 105.5, 103.0, 92.9, 70.6, 70.3, 70.0, 69.3, 69.1, 69.0, 68.6, 14.7.

**Preparation of receptor B2.** To a solution of compound **4** (0.45 g, 1.0 mmol) in methanol (20 mL) was added aniline (0.92 mL, 1.0 mmol), and the mixture was stirred for 24 h at room temperature. Then the reaction was concentrated and purified by silica gel column chromatography (5% methanol in dichloromethane) to give receptor B2 as an orange solid (0.3 g, 0.59 mmol, 59%). HRMS (ESI): calcd for C_29_H_24_BF_2_N_4_O_2_ [M − H]^−^ 509.1966; found: 509.1980. ^1^H NMR (300 MHz, DMSO-d_6_, δ ppm): 10.20 (s, 1H), 10.08 (s, 1H), 7.75 (d, J 9 Hz, 2H), 7.55 (d, J 9 Hz, 2H), 7.54–7.35 (m, 4H), 7.11 (m, 1H), 6.20 (s, 2H), 2.46 (s, 6H), 1.46 (s, 6H). ^13^C NMR (75 MHz, DMSO-d_6_, δ ppm): 182.3, 182.0, 166.7, 166.1, 165.8, 155.3, 143.2, 142.2, 140.2, 140.1, 139.5, 139.5, 139.0, 138.9, 131.4, 130.0, 129.9, 129.8, 129.6, 128.8, 124.0, 123.9, 121.8, 119.1, 119.0, 118.9, 14.8.

## 4. Conclusions

In conclusion, we successfully developed a ditopic ion-pair sensor, B1, and compared its binding properties with those of compounds B2, acting as an anion sensor, and B3, stripped of the ion-binding domains. The cooperativity in ion-pair binding by the B1 receptor was demonstrated during titration studies using UV–Vis and ^1^H NMR spectroscopy in CH_3_CN. The formation of complexes of B1 with salts and the specific distribution of ions within the binding domains was also confirmed by X-ray analysis. B1’s ability to form J aggregates in an aqueous environment and the prospect of visual salt detection in this medium were demonstrated. B1 was determined to be effective in transporting potassium chloride through a bulk liquid organic membrane (U-tube). Conversely, B1 was able to be transported through an aqueous membrane consisting of KCl salt, thus leading to inverted U-tube experiments. The presence of the BODIPY reporter in ion-pair sensor B1’s structure allowed for the creation of a new unique ON1-OFF-ON2-ON3 fluorescent response due to the interaction with fluoride ions. Moreover, a four-step output mechanism was proposed and studied, consisting of the initial B1 emission (ON1), the (eT) electron-transfer process (OFF), the deprotonation event (ON2), and the disruption of the stable difluoroboron bridge in the BODIPY unit (ON3).

## Figures and Tables

**Table 1:** Association constants (K_a_) for interactions between B1 and selected anions in the absence or presence of 1 equivalent of KPF_6_^a^.

TBA^+^	B1	B1 + 1 equiv. K^+^	K_K_^+^_/TBA_^+^
Cl^−^	1.60 × 10^5^	3.40 × 10^5^	2.1
Br^−^	0.95 × 10^4^	2.15 × 10^4^	2.3
NO_2_^−^	4.61 × 10^4^	6.96 × 10^4^	1.5
NO_3_^−^	1.72 × 10^4^	2.51 × 10^4^	1.5

^a^ UV–Vis, solvent CH_3_CN, temperature 293 K, [B1] = 1.0 × 10^−5^ M. All anions were used in the form of their respective tetrabutylammonium (TBA) salts. [TBAX] ≈ 0.8 × 10^−3^ M; M^−1^, potassium added as KPF_6_, Errors < 10%.

## Data Availability

All study data are included in the main text and Supplementary Materials.

## Supplementary Materials

The supporting information can be downloaded at: https://www.mdpi.com/article/10.3390/ijms24108536/s1. References [58,59,60,61,62,63,64,65,66,67] are cited in the supplementary materials.
