# Supplementary material for: Interaction of Ions in Organic and Aqueous Media with an Ion-Pair Sensor Equipped with a BODIPY Reporter: An ON1-OFF-ON2-ON3 Fluorescent Assay
Source: Int J Mol Sci. 2023 May 10;24(10):8536. doi: 10.3390/ijms24108536 (PMC10218023; doi:10.3390/ijms24108536)
Supplement: Supplementary file 1 [file ijms-24-08536-s001.zip › ijms-2370138-supplementary.pdf]

Interaction of Ions in Organic and Aqueous Media With an Ion Pair Sensor Equipped With a BODIPY Reporter: An ON1-OFF-ON2-ON3 Fluorescent Assay

Marta Zaleskaya-Hernik, Łukasz Dobrzycki and Jan Romański *

Faculty of Chemistry, University of Warsaw, Pasteura 1, PL 02-093 Warsaw, Poland

*jarom@chem.uw.edu.pl

Table of Contents

1. **General information** **S2**
2. **NMR spectra** **S3**
3. **UV-vis titration experiments** **S6**
4. **NMR titration** **S14**
5. **Crystal data** **S17**
6. **NMR experiments** **S22**
7. **Dynamic light scattering measurements (DLS)** **S30**
8. **Extraction experiments - ion chromatography** **S31**
9. **Transport Studies** **S32**
10. **Detection limit** **S33**
11. **Fluorescent experiments** **S35**
12. **References** **S40**

General information

Unless specifically indicated, all other chemicals and reagents used in this study were purchased from commercial sources and used as received. If necessary purification of products was performed using column chromatography on silica gel (Merck Kieselgel 60, 230-400 mesh) with mixtures of chloroform/methanol. Thin-layer chromatography (TLC) was performed on silica gel plates (Merck Kieselgel 60 F254).

^{1}H and ^{13}C NMR spectra used in the characterization of products were recorded on Bruker 300 spectrometer using a residual protonated solvent as internal standard. DOSY experiments were conducted at 298 K on Varian VNMRS 600 MHz instruments with a residual solvent signal as an internal standard.

Mass spectra were measured on Quattro LC Micromass or Shimadzu LCMS-IT-TOF unit.

UV-vis analyses were performed using Thermo Spectronic Unicam UV500 Spectrophotometer.

Fluorescence emission spectra were measured on a Hitachi F-7100 Fluorescence Spectrophotometer at 298K.

High performance ion chromatography (HPIC) analyses were performed using a 930 Compact apparatus.

NMR spectra

^{1}H and ^{13}C NMR spectra were recorded on a Bruker 300 MHz spectrometer. ^{1}H NMR chemical shifts δ are reported in ppm referenced to residual solvent signal (DMSO-d_6).

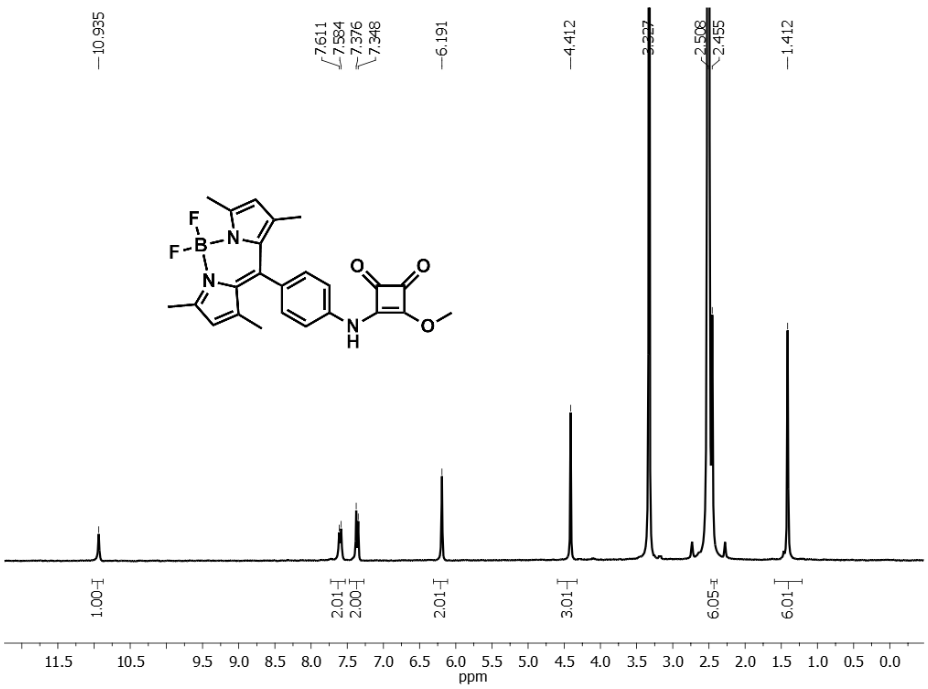

Figure S1. ^{1}H NMR spectrum of compound 4 in DMSO-d_6.

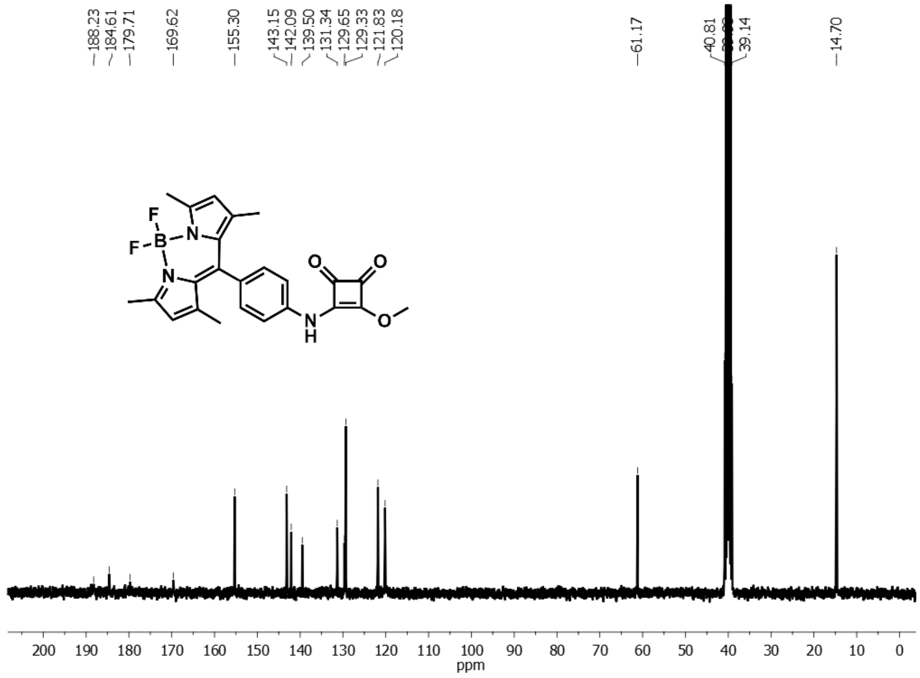

Figure S2. ^{13}C NMR spectrum of compound 4 in DMSO-d_6.

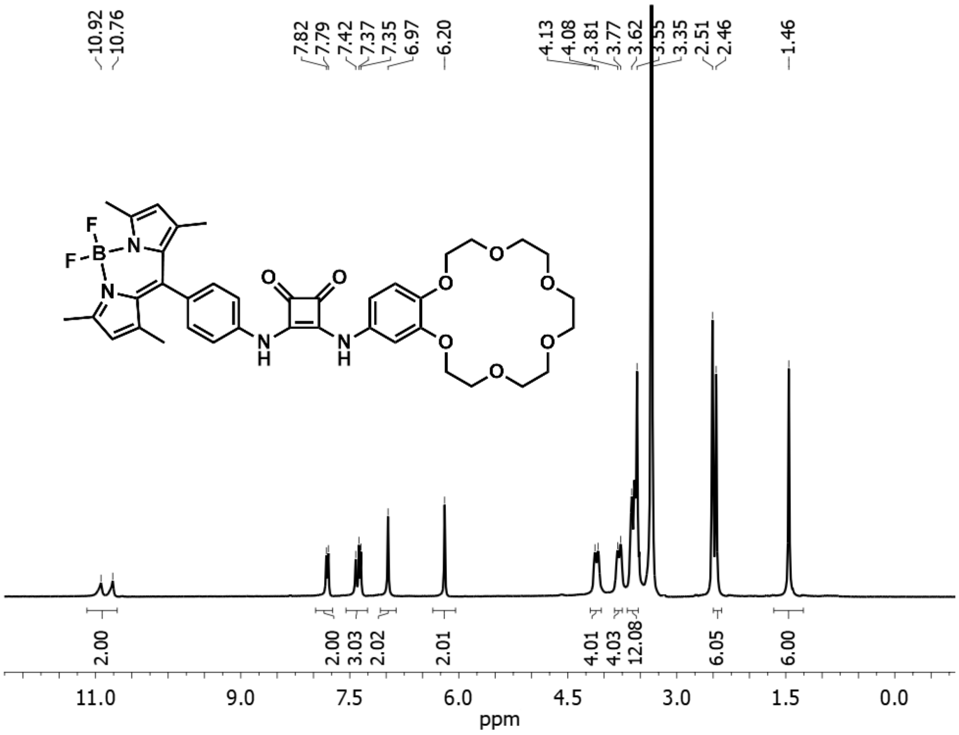

Figure S3. ^{1}H NMR spectrum of receptor B1 in DMSO-d_6.

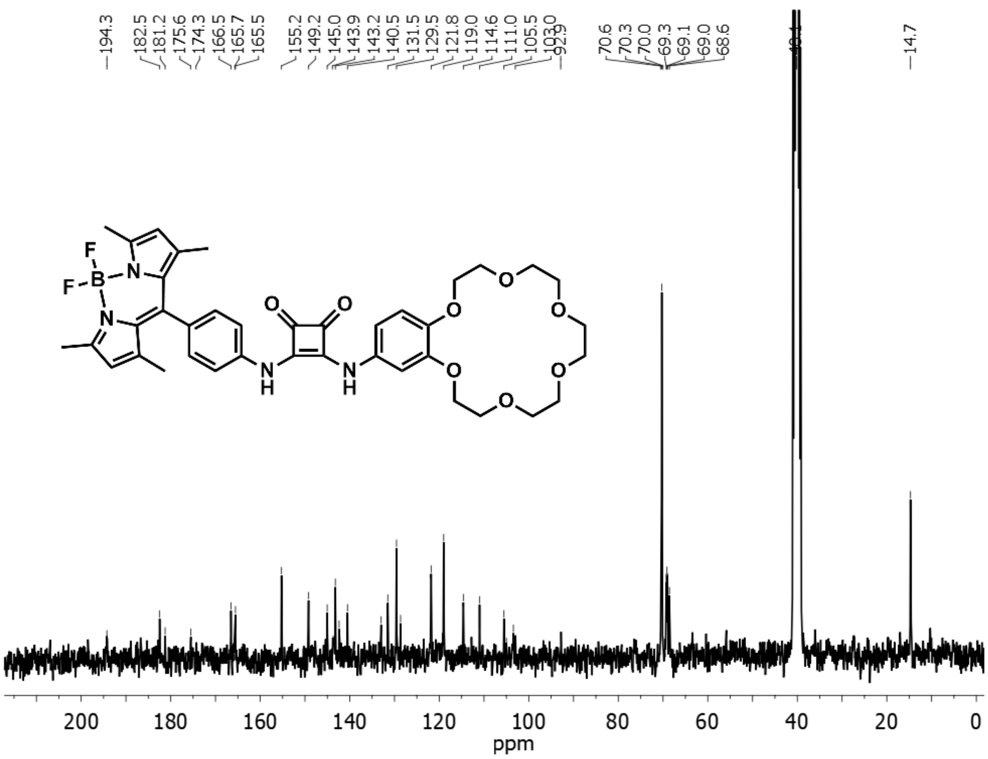

Figure S4. ^{13}C NMR spectrum of receptor B1 in DMSO-d_6.

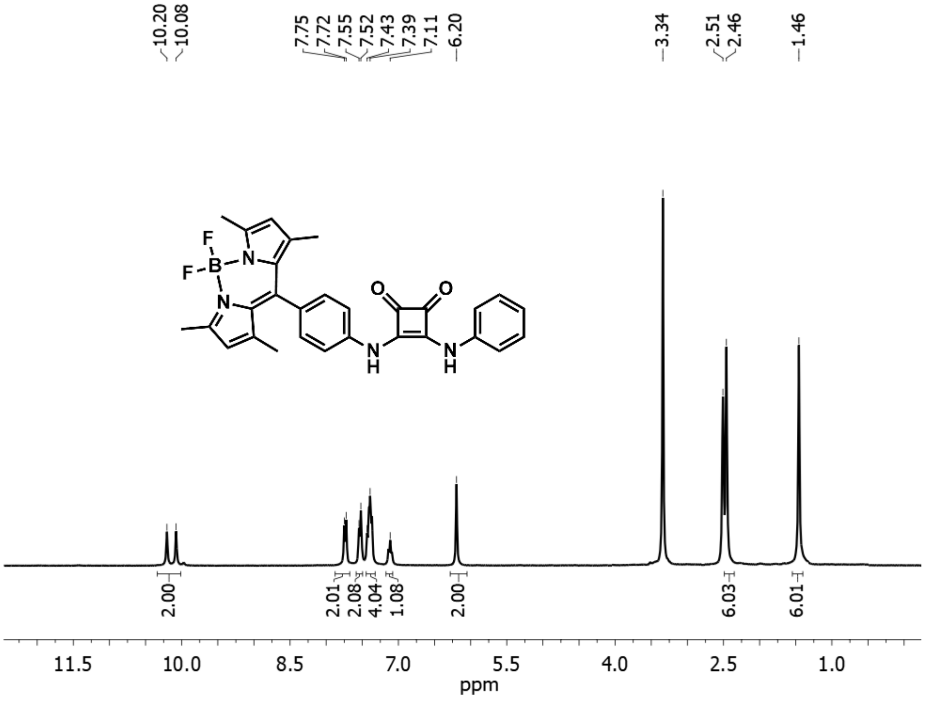

Figure S5. ^{1}H NMR spectrum of receptor B2 in DMSO-d_6.

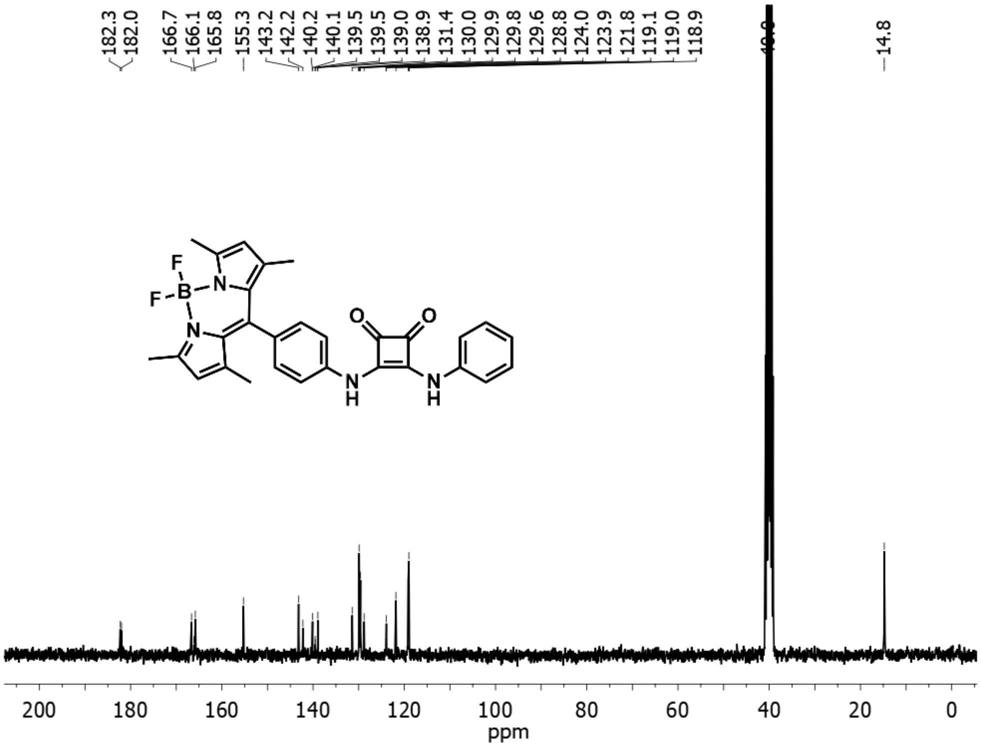

Figure S6. ^{13}C NMR spectrum of receptor B2 in DMSO-d_6.

UV-vis titration experiments

UV–vis titrations: experiments were performed in CH_3CN solution at 298K using a Hitachi U-2910 spectrophotometer. To 10 mm cuvette was added 2.5 mL of freshly prepared (B1 – c = 1.0 ×10^{-5} M; B2 – c = 1.1 ×10^{-5} M) solution of studied receptor and in case of ion-pair binding studies, 1 mol equivalent of cation (KPF_6 or $NaClO_4$) was added before titrations. Small aliquots of ca. 1.0 ×10^{-3} M TBAX solution containing suitable the receptor at the same concentration as in cuvette were added and a spectrum was acquired after each addition. The resulting titration data were analysed using BindFit (v0.5) package, available online at http://supramolecular.org. Each titration was carried out in duplicate. Reported values are calculated as weighted arithmetic mean, where the weights were the errors obtained for each value separately. The given uncertainty of the association constants is the largest of the variance (external or internal).

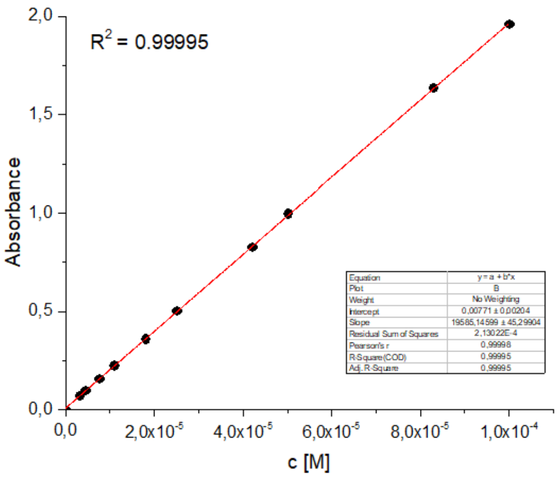

Figure S7. Dilution curve of B1 in CH_3CN.

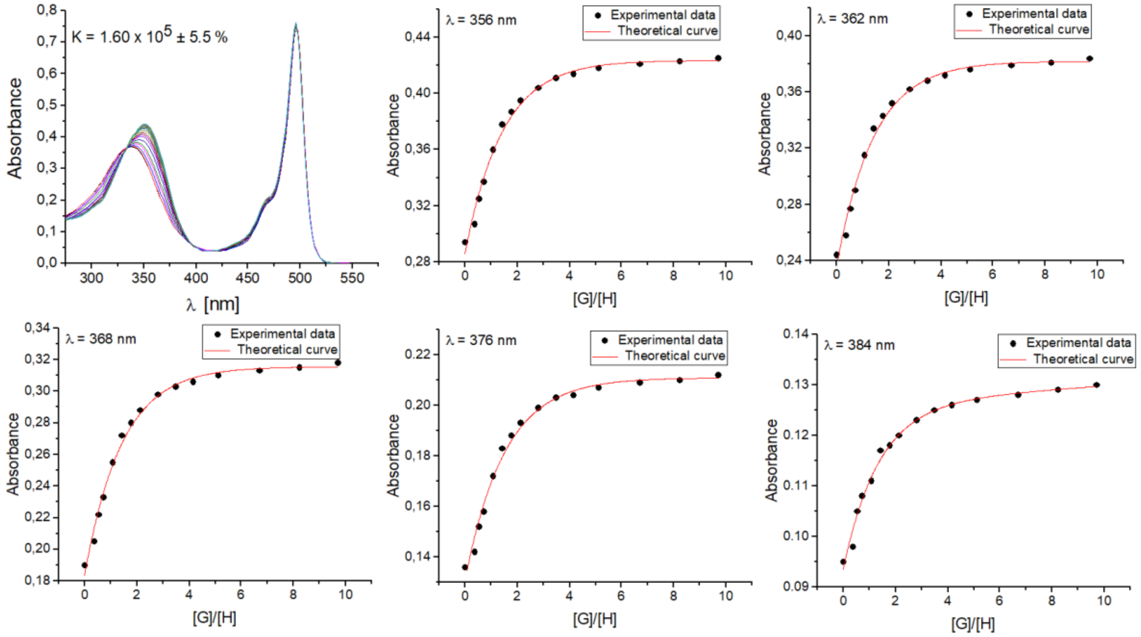

Figure S8. UV-vis titration of B1 with TBACl in CH_3CN and selected binding isotherms.

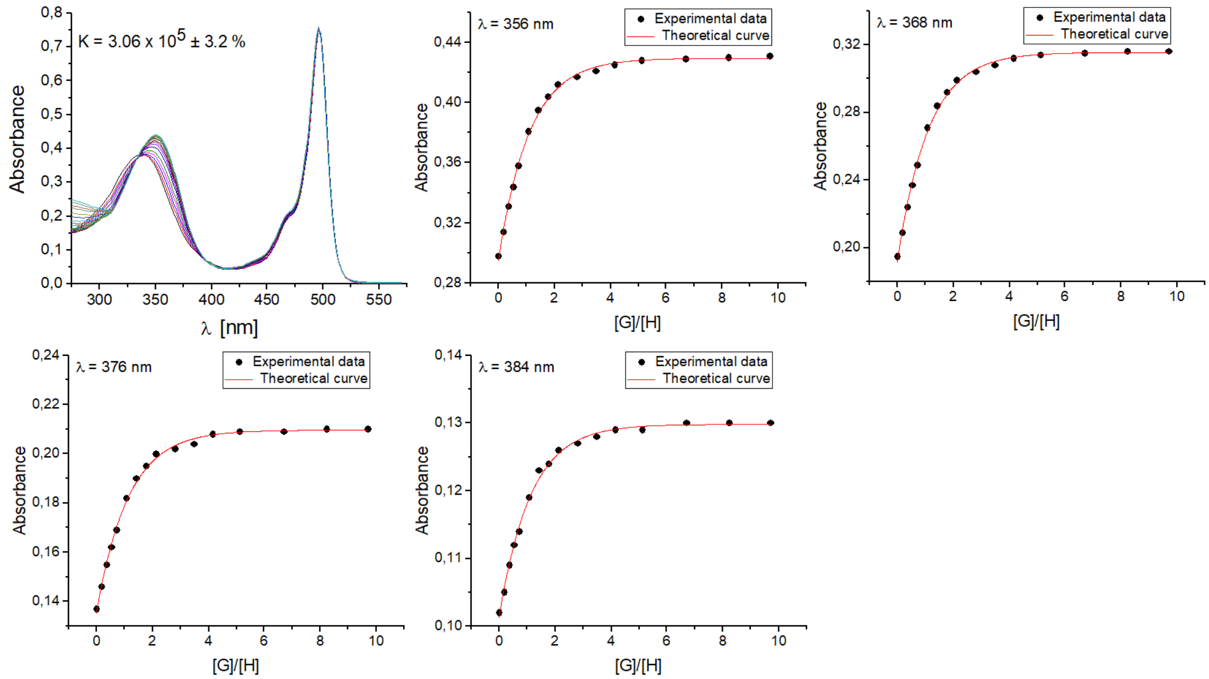

Figure S9. UV-vis titration of B1 with TBACl in the presence of 1 equivalent of $NaClO_4$ in CH_3CN and selected binding isotherms.

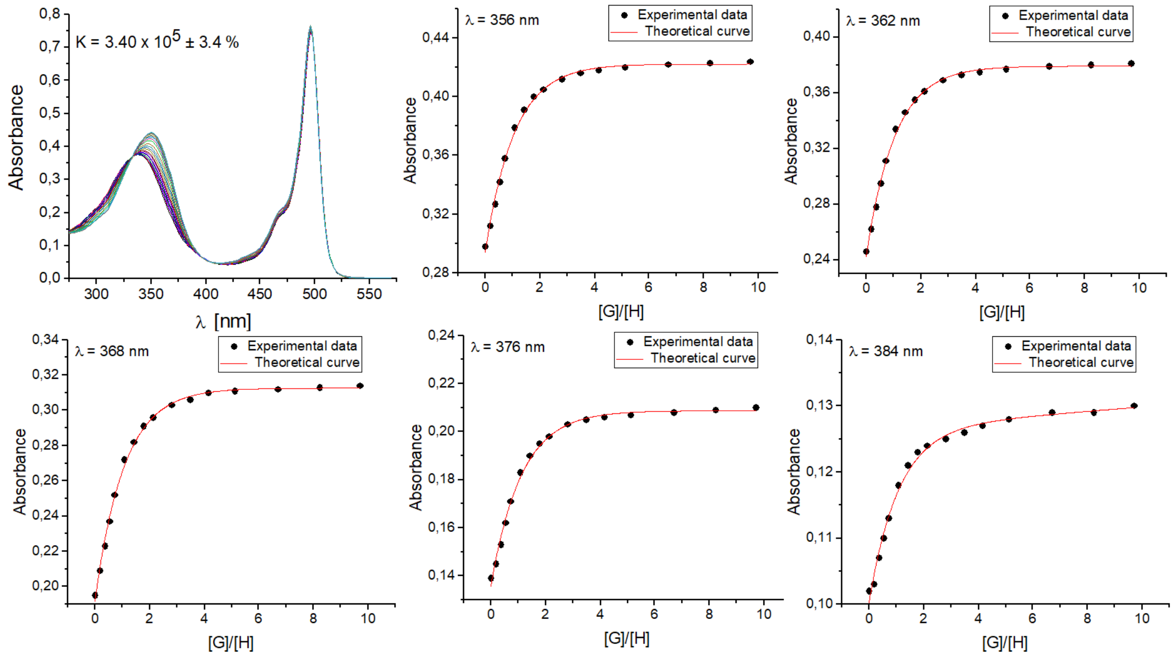

Figure S10. UV-vis titration of B1 with TBACl in the presence of 1 equivalent of KPF_6 in CH_3CN and selected binding isotherms.

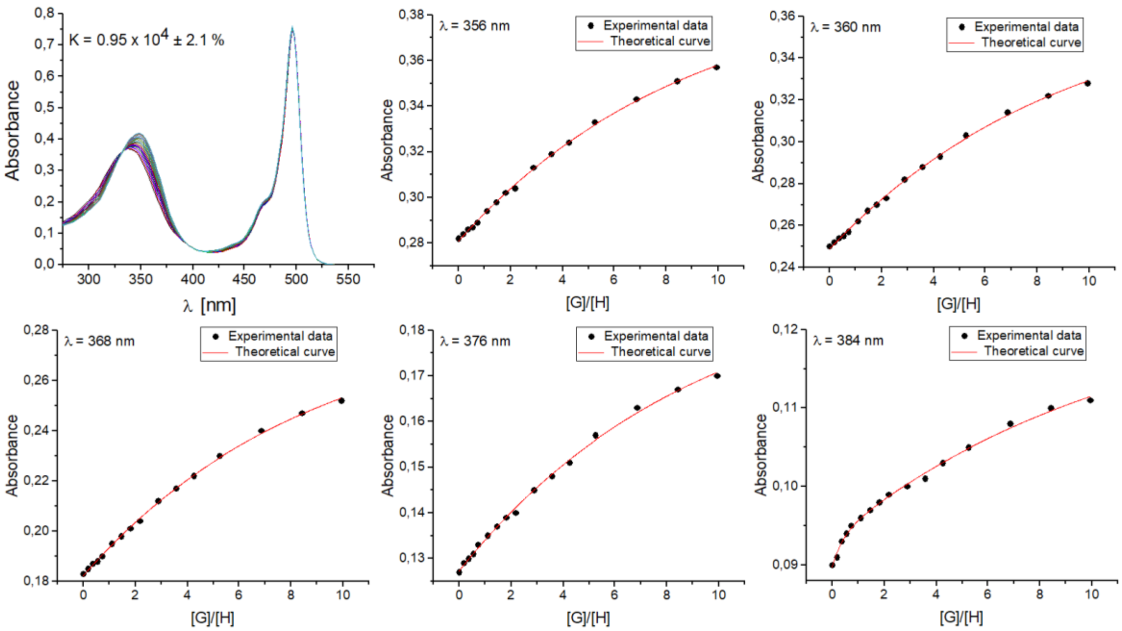

Figure S11. UV-vis titration of B1 with TBABr in CH_3CN and selected binding isotherms.

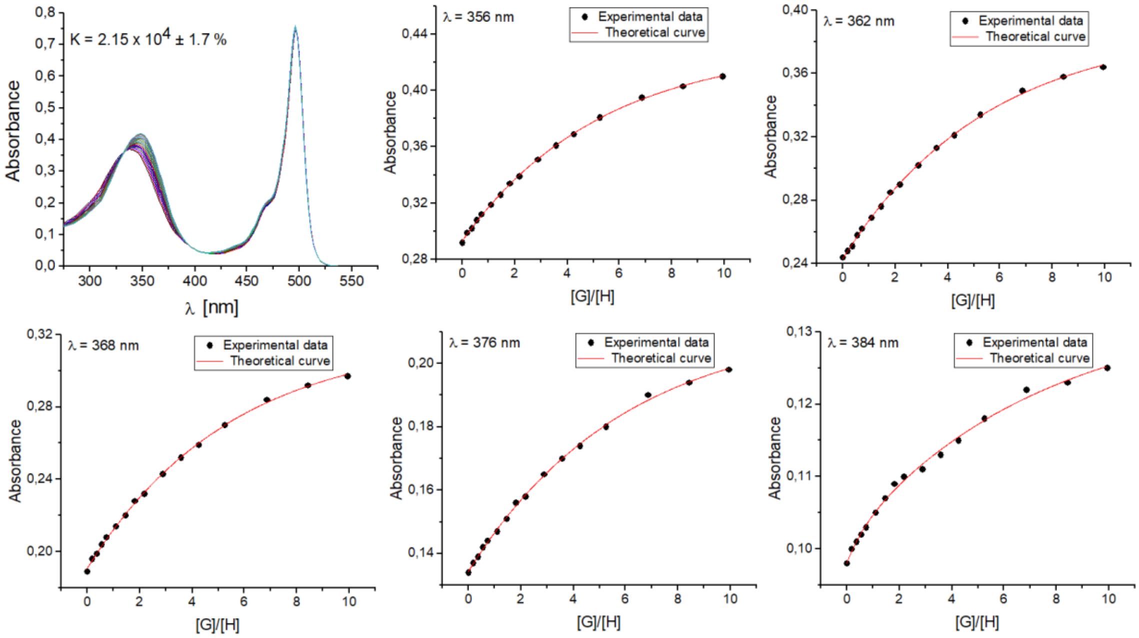

Figure S12. UV-vis titration of B1 with TBABr in the presence of 1 equivalent of KPF_6 in CH_3CN and selected binding isotherms.

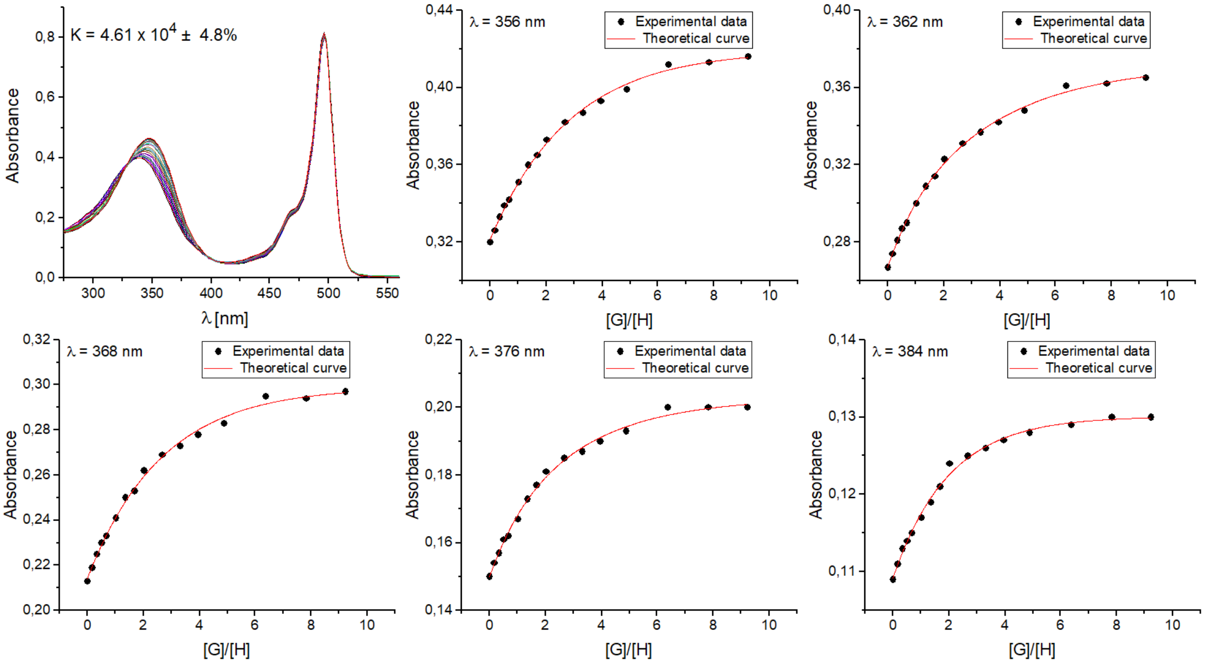

Figure S13. UV-vis titration of B1 with $TBANO_2$ in CH_3CN and selected binding isotherms.

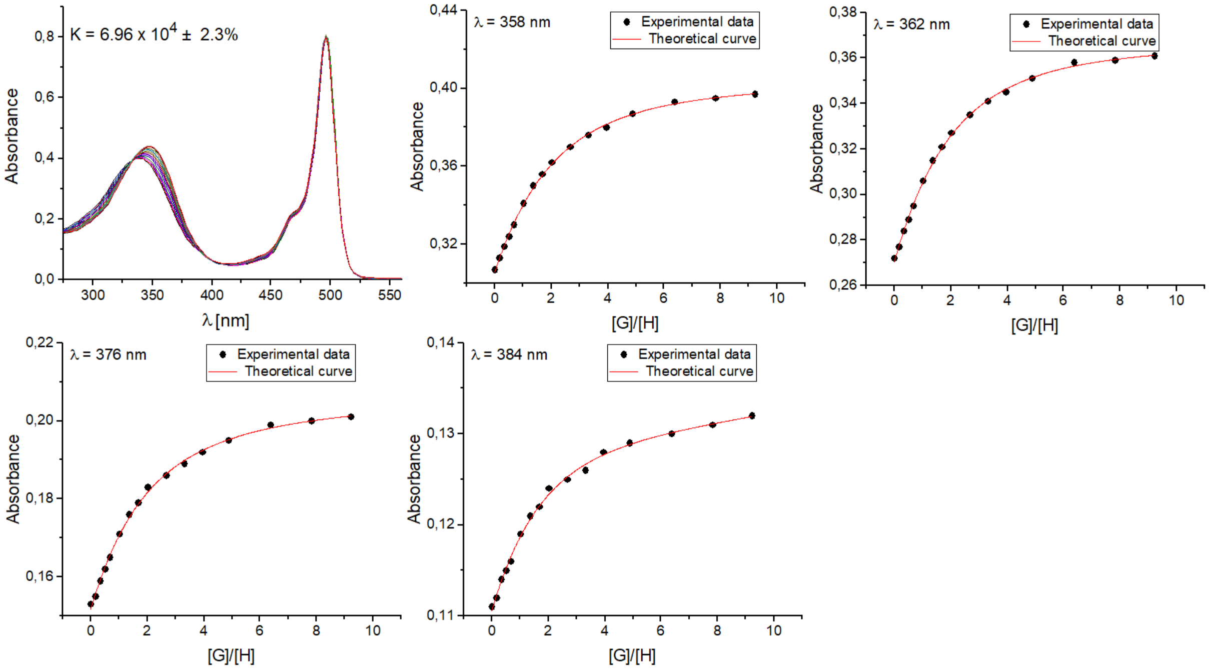

Figure S14. UV-vis titration of B1 with $TBANO_2$ in the presence of 1 equivalent of KPF_6 in CH_3CN and selected binding isotherms.

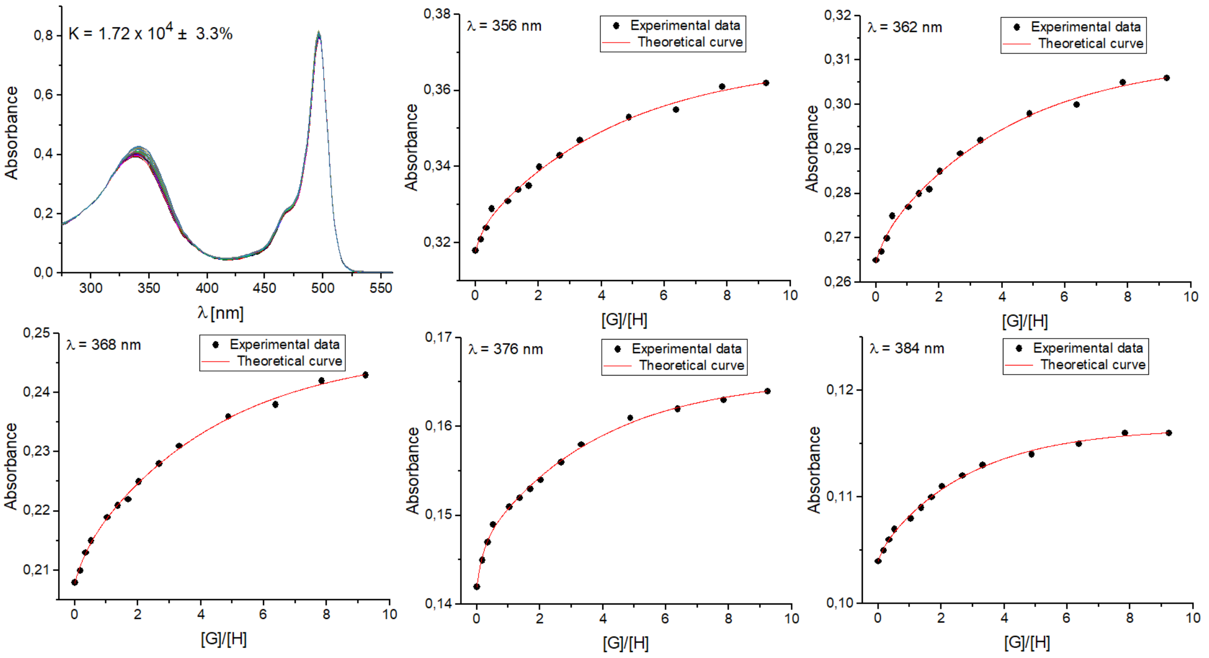

Figure S15. UV-vis titration of B1 with $TBANO_3$ in CH_3CN and selected binding isotherms.

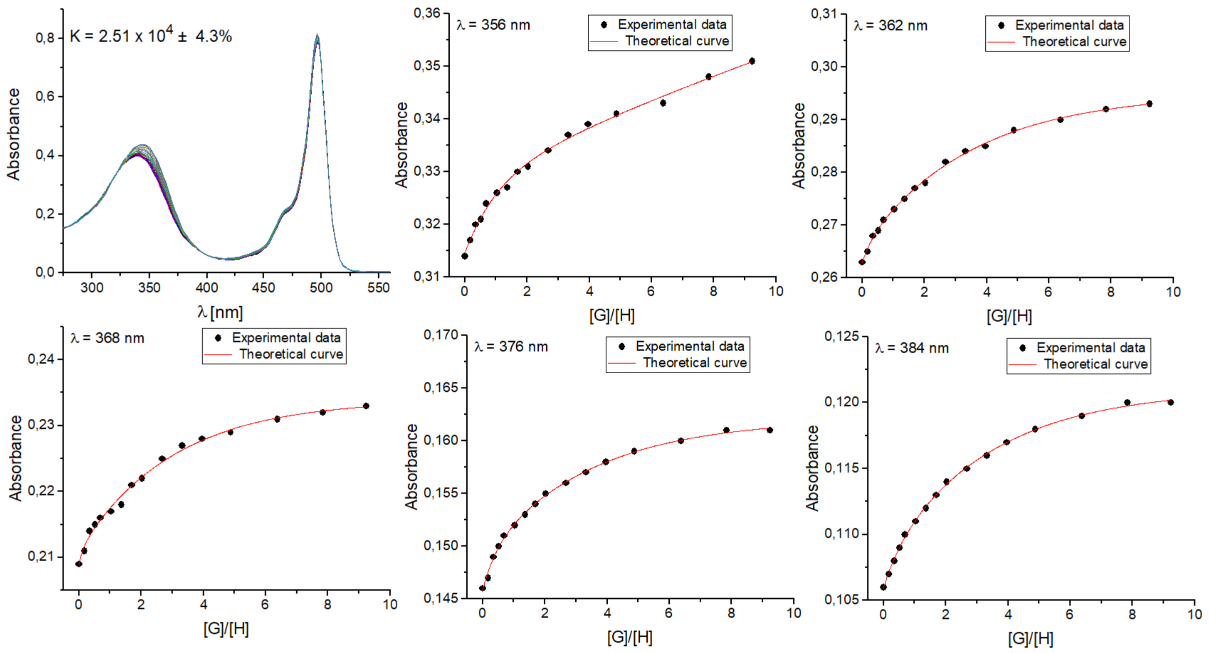

Figure S16. UV-vis titration of B1 with $TBANO_3$ in the presence of 1 equivalent of KPF_6 in CH_3CN and selected binding isotherms.

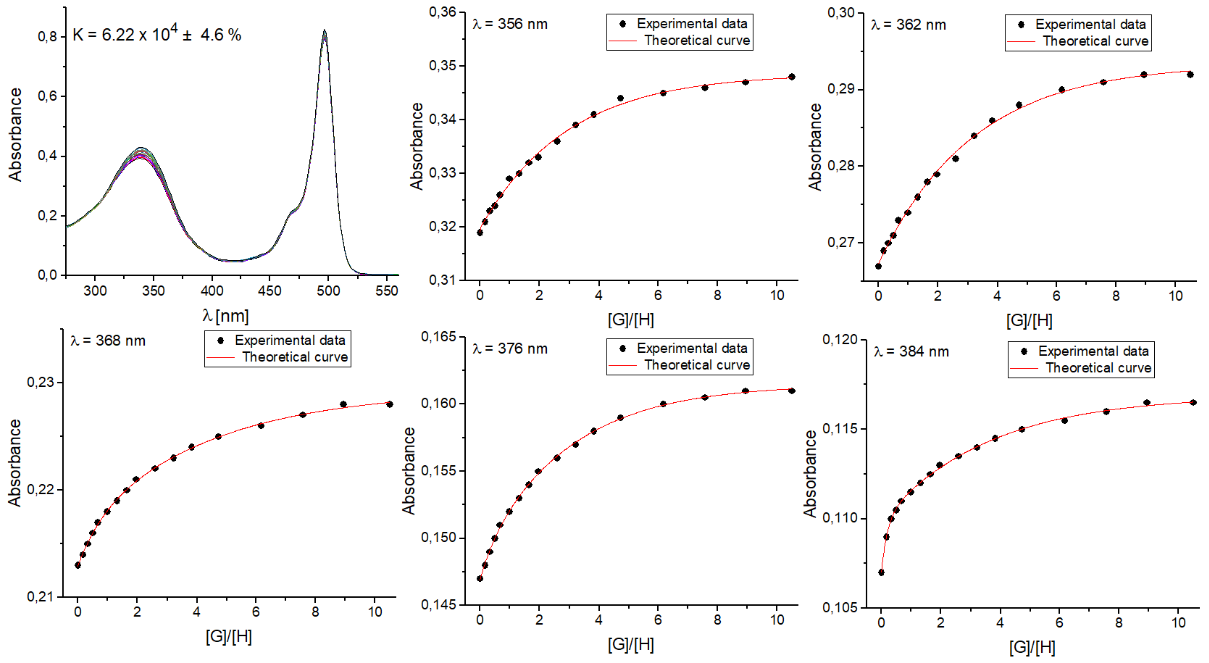

Figure S17. UV-vis titration of B1 with KPF_6 in CH_3CN and selected binding isotherms.

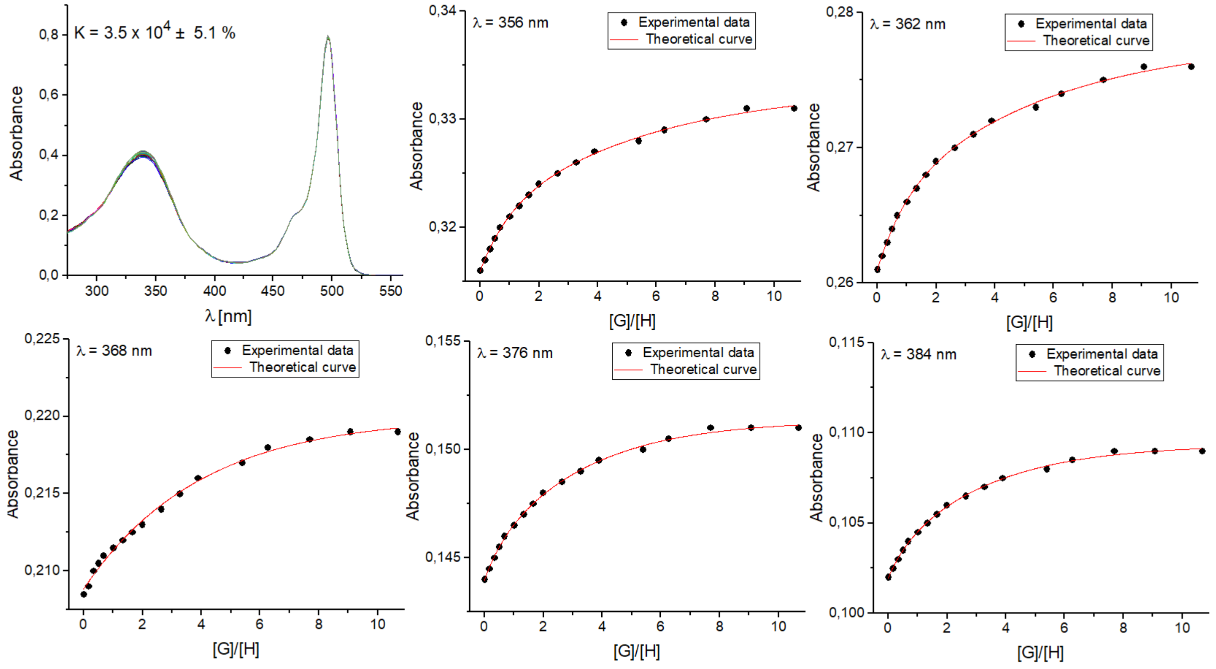

Figure S18. UV-vis titration of B1 with $NaClO_4$ in CH_3CN and selected binding isotherms.

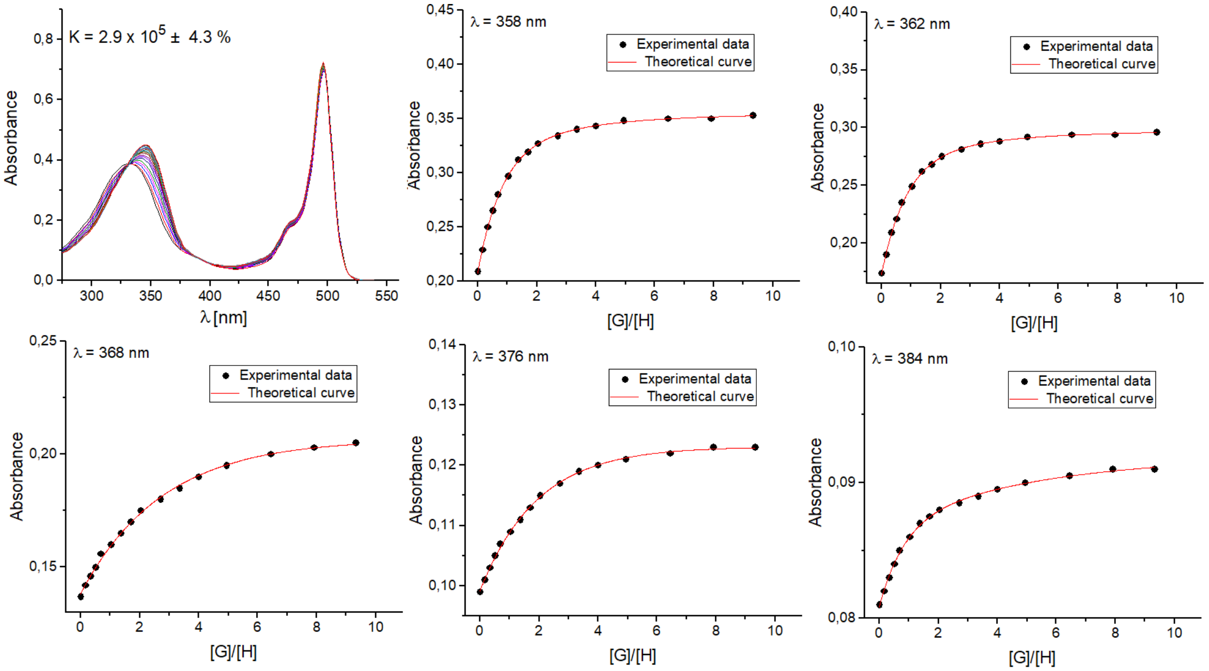

Figure S19. UV-vis titration of B2 with TBACl in CH_3CN and selected binding isotherms.

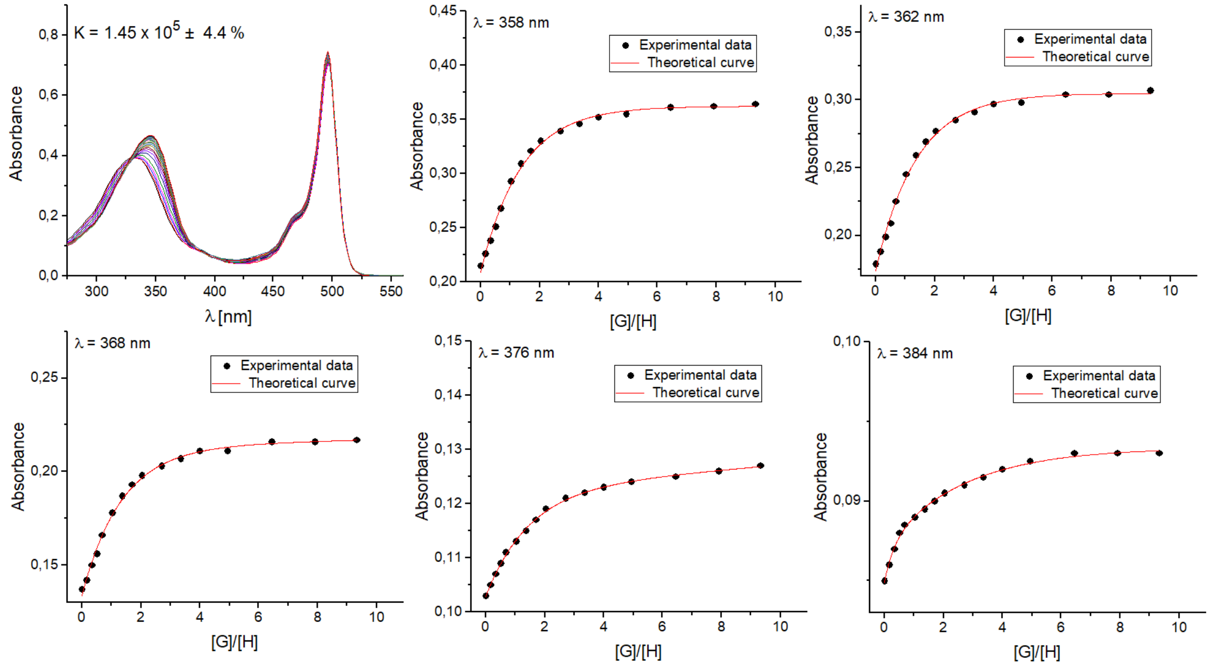

Figure S20. UV-vis titration of B2 with TBACl in the presence of 1 equivalent of $NaClO_4$ in CH_3CN and selected binding isotherms.

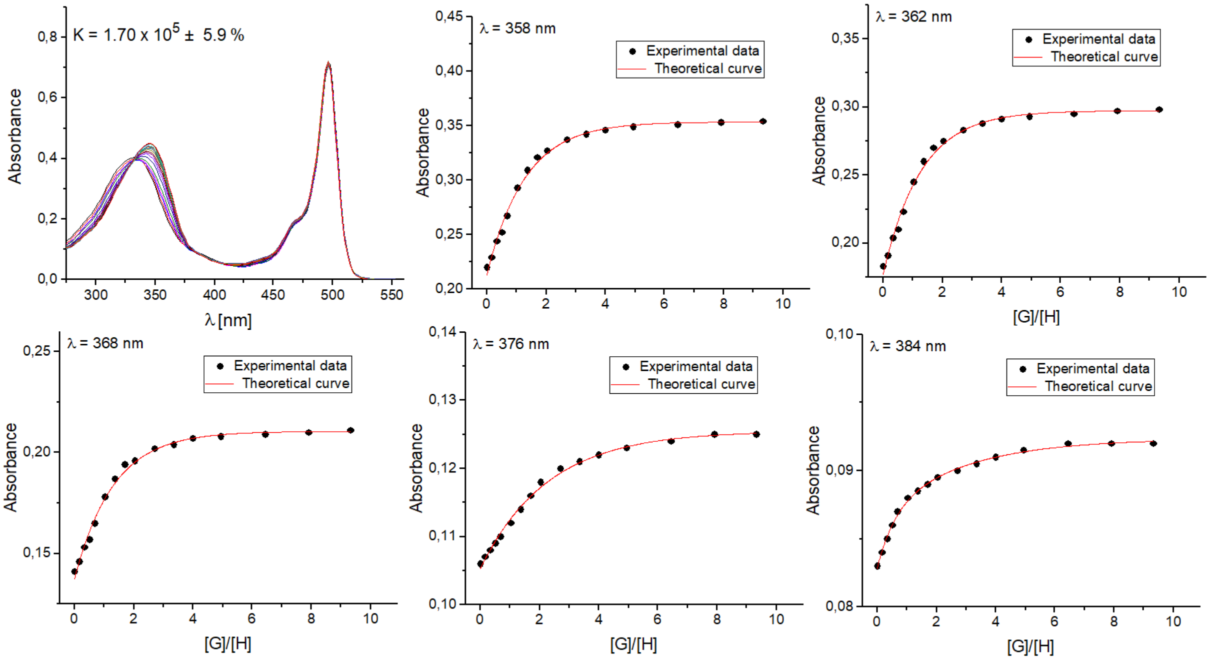

Figure S21. UV-vis titration of B2 with TBACl in the presence of 1 equivalent of KPF_6 in CH_3CN and selected binding isotherms.

NMR titration

The ^{1}H NMR titration was conducted at 298K in DMSO-*d6*. In each case, a 500 μL of freshly prepared 1.9 mM solution of B1 was added to a 5 mm NMR tube. In the case of ion pair titration receptor B1 was firstly pretreated with one equivalent of KPF_6. Then small aliquots of solution of TBAX, containing receptor at constant concentration, were added and a spectrum was acquired after each addition. The resulting titration data were analyzed using BindFit (v0.5) package, available online at http:// supramolecular.org.

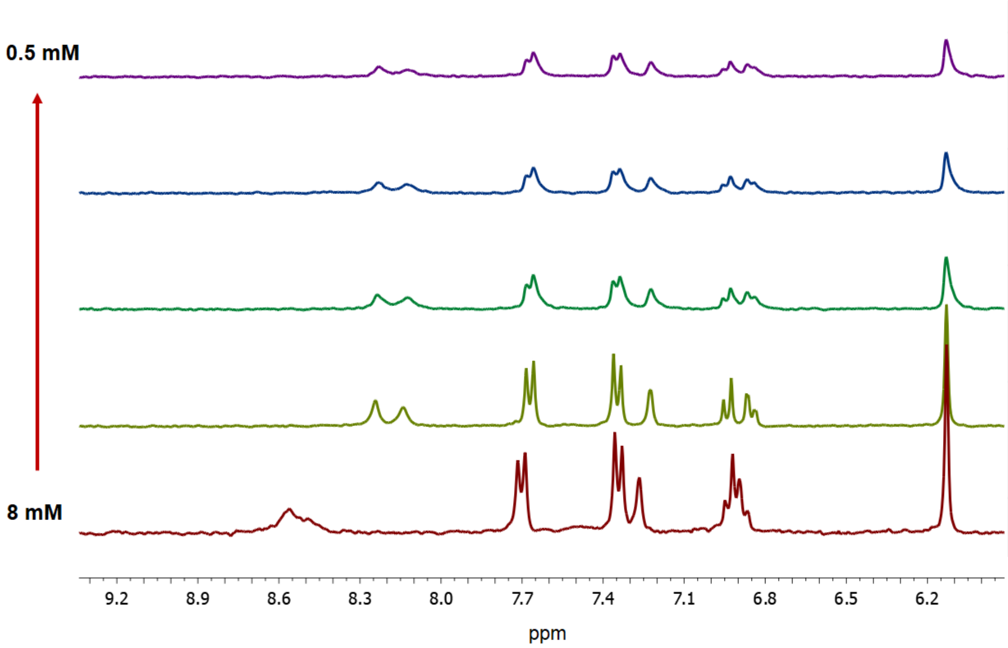

Figure S22. ^{1}H NMR spectra recorded upon dilution of B1 in CD_3CN.

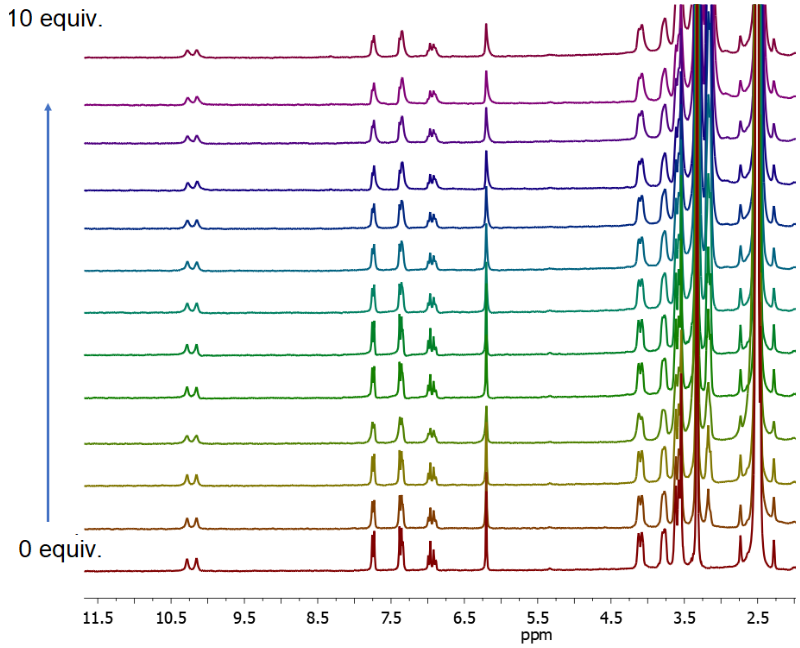

Figure S23. Partial ^{1}HNMR spectra recorded upon titration of **B1** in DMSO-*d6* with $TBANO_3$.

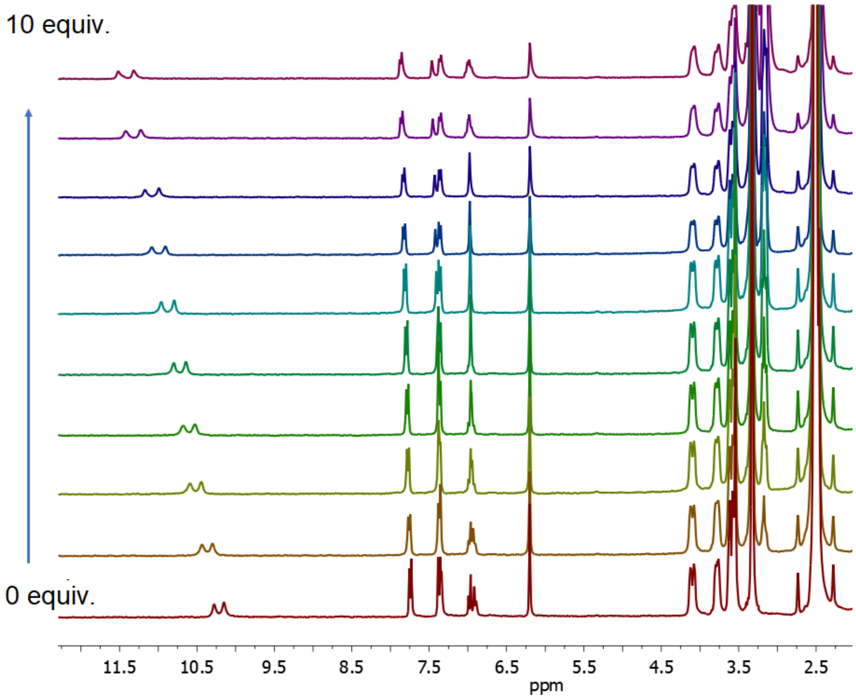

Figure S24. Partial ^{1}HNMR spectra recorded upon titration of **B1** in DMSO-*d6* with TBACl.

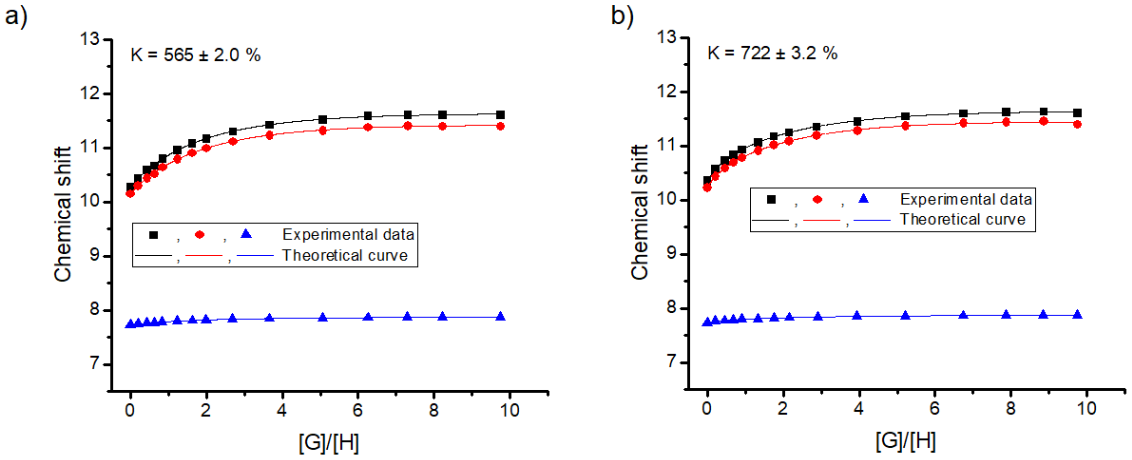

Figure S25. ^{1}H NMR titration binding isotherms of **B1** in DMSO-*d6* (a) upon addition of increasing amounts of TBACl, (b) in the presence of 1 equivalent of KPF_6.

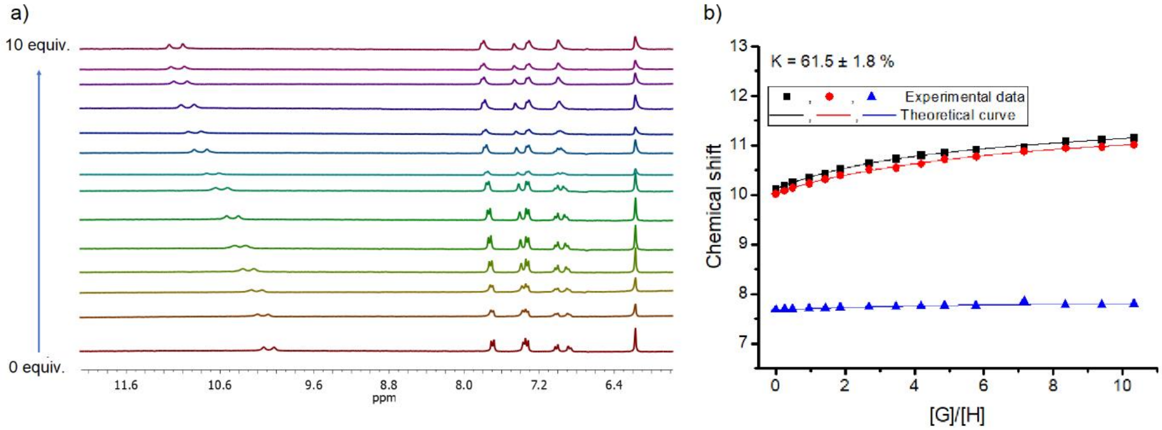

Figure S26. a) Partial ^{1}HNMR spectra recorded upon titration of **B1** (2.0 mM) with TBACl (signals corresponding to phenyl protons) namely in the presence of a 10% aqueous 0.5M KNO_3 in the DMSO-*d6* solution and binding isotherms, b) ^{1}H NMR titration binding isotherms.

Crystal data

The X-ray measurement of **B1_KBr** was performed at 130.0(5) K on a Bruker D8 Venture PhotonII diffractometer equipped with a mirror monochromator and a CuKα INCOATEC IμS micro-focus source (λ = 1.54178 Å). A total of 4340 frames were collected with Bruker APEX3 program.[S1] The frames were integrated with the Bruker SAINT software package[S2] using a narrow-frame algorithm. The integration of the data using a triclinic unit cell yielded a total of 31211 reflections to a maximum θ angle of 66.55° (0.84 Å resolution), of which 7755 were independent (average redundancy 4.025, completeness = 99.5%, R_{int} = 3.41%, R_{sig} = 2.88%) and 7020 (90.52%) were greater than 2σ(F^2). The final cell constants of a = 13.1099(18) Å, b = 13.7136(18) Å, c = 14.429(2) Å, α = 108.792(5)°, β = 92.826(5)°, γ = 113.386(4)°, V = 2206.7(5) Å^3, are based upon the refinement of the XYZ-centroids of 9808 reflections above 20 σ(I) with 6.609° < 2θ < 133.4°. Data were corrected for absorption effects using the Multi-Scan method (SADABS).[S3] The ratio of minimum to maximum apparent transmission was 0.887. The calculated

minimum and maximum transmission coefficients (based on crystal size) are 0.605 and 0.944.

The structure was solved and refined using SHELXTL Software Package [S4,S5] using the space group $P\bar{1}$, with Z = 1 for the formula unit, $C_{86.63}H_{111.61}N_8O_{20.17}B_2F_4Br_2K_{0.17}Na_{1.83}$ corresponding to: 2×($C_{39}H_{43}N_4O_8B_1F_2$) + 2×($K_{0.085}Na_{0.915}Br$) + 1.829×(MeOH) + 0.171×(H_2O) + 1.544×(Et_2O) + 0.628×(MeOH). The final anisotropic full-matrix least-squares refinement on F^2 with 656 variables converged at $R1$ = 3.81%, for the observed data and $wR2$ = 9.82% for all data. The goodness-of-fit was 1.120. The largest peak in the final difference electron density synthesis was 0.418 $e^-/Å^3$ and the largest hole was -0.420 $e^-/Å^3$ with an RMS deviation of 0.056 $e^-/Å^3$. On the basis of the final model, the calculated density was 1.425 g/cm^3 and $F(000)$, 988 e^-. The details concerning the crystal data and structural parameters of **B1_KBr** are collected in **Table S2**.

The structure contains mixed KBr/NaBr salt and severely disordered Et_2O and MeOH species.

The refined occupancy K:Na ratio yields 0.085(1):0.915(1). The presence of different cation type is associated with different orientation of the squaramide unit of the ligand. In case of Na^+ ion presence the cation located in the crown ether ring is additionally stabilized by the MeOH molecule with the occupancy equal 0.915(1). The OH group of the methanol forms hydrogen bond with the partially occupied [0.915(1)] Br^- anion. In such a configuration additional disordered Et_2O and MeOH molecules are present in the structure with the latter forming hydrogen bond with carbonyl atom of the squaramide moiety. In the site containing Et_2O/MeOH species refined occupancy ratio yields 0.350(3):0.255(3):0.167(3):0.228(3) with the first three values corresponding to alternative positions of diethyl ether whereas the last value stands for partial occupancy methanol moiety. In case of the K^+ presence the cation sitting in the crown ether ring, and sharing the same position with Na^+ ion, is coordinated by partial occupancy H_2O molecule

[occupancy equal 0.085(1)]. Different orientation of the squaramide moiety [all corresponding atoms with refined partial occupancy of 0.085(1)] causes different location of the partial occupancy Br^- ion and presence of 0.0815(1) of MeOH instead of Et_2O/MeOH moieties. The different distribution of moieties in the crystal structure of **B1_KBr** depending on the cation type is presented in Figure S27. To preserve reasonable geometry of the disordered moieties a number of geometry restraints was used supported by modelling thermal parameters of low occupancy atoms. All ordered and >50% occupancy disordered non-H atoms were refined anisotropically. Most of hydrogen atoms were placed in calculated positions and refined within the riding model. Positions of hydrogen atoms of hydroxyl groups of MeOH and H_2O molecules were refined with distance restraints to neighboring heavy atoms. The temperature factors of hydrogen atoms were not refined and were set to be either 1.2 or 1.5 times larger than U_{eq} of the corresponding heavy atom. The atomic scattering factors were taken from the International Tables.[S6] Molecular graphics was prepared using program Mercury 2020.2.0.[S7] Thermal ellipsoids parameters are presented in Figure S28 at 50% probability level.

Table S2. Data collection and structure refinement for **B1_KBr**.

**Identification code**	**B1_KBr**
**Formula**	$C_{86.63}H_{111.61}N_8O_{20.17}B_2F_4Br_2K_{0.17}Na_{1.83}$ corresponding to: $2\times(C_{39}H_{43}N_4O_8B_1F_2) + 2\times(K_{0.085}Na_{0.915}Br) + 1.829\times(CH_4O) + 0.171\times(H_2O) + 1.544\times(C_4H_{10}O) + 0.628\times(CH_4O)$
**M_x/ g·mol^{-1}**	1893.88
**T/ K**	130.0(5)
**λ/ Å**	1.54178
**Crystal size**	0.029×0.081×0.276 mm
**Space group**	$P\bar{1}$
**Unit cell dimensions**	a = 13.1099(18) Å α = 108.792(5)°<br>b = 13.7136(18) Å β = 92.826(5)°<br>c = 14.429(2) Å γ = 113.386(4)°
**V/ Å^3, Z**	2206.7(5), 1
**D_x/ g·cm^{-3}**	1.425
**μ/ mm^{-1}**	2.027

**F(000)**	988
**θ_{min}, θ_{max}**	3.31°, 66.55°
**Index ranges**	$-15 \le h \le 15$<br>$-16 \le k \le 16$<br>$-16 \le l \le 17$
**Reflections collected/ independent**	31211/ 7755<br>(R_{int} = 0.0341)
**Completness**	99.5%
**Absorption correction**	Multi-Scan
**T_{max}, T_{min}**	0.944 and 0.605
**Refinement method**	Full-matrix LSQ on F^2
**Data / restraints / parameters**	7755 / 69 / 656
**Gof on F^2**	1.120
**Final R indices**	7020 data; I>2σ(I)<br>R1 = 0.0381, wR2 = 0.0926<br>all data<br>R1 = 0.0434, wR2 = 0.0982
**$\Delta\rho_{max}$, $\Delta\rho_{min}$**	0.418, -0.420 e·Å^{-3}

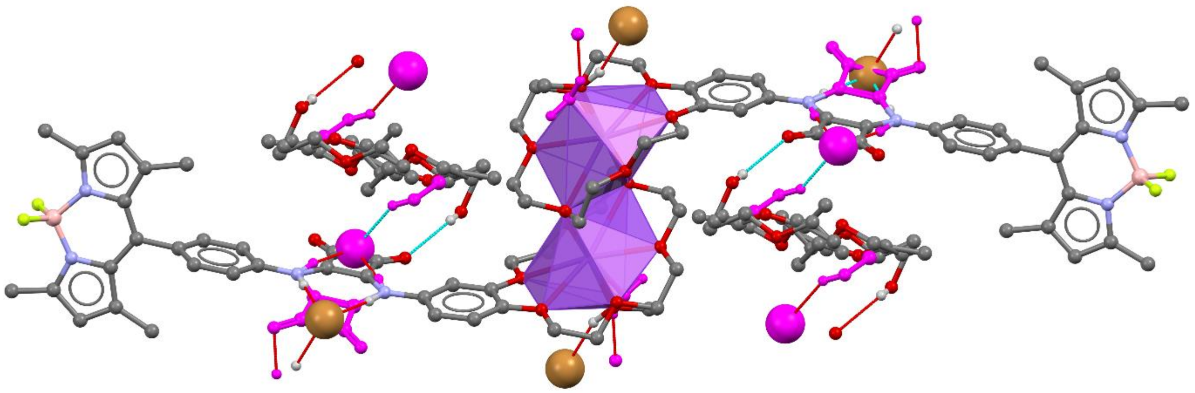

Figure S27. Different distribution of species in the structure depending on the cation type. The moieties coloured in magenta corresponds to presence of 0.085(1) of K^+ cation. Large brown/magenta spheres stand for Br^- ions.

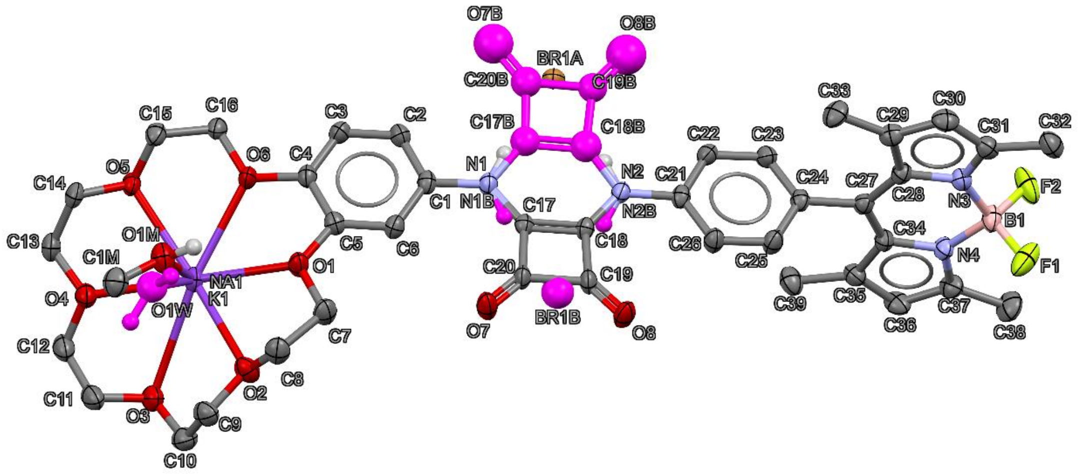

Figure S28. Numbering scheme and thermal displacement ellipsoids of non-H atoms presented at 50% probability level. Disordered Et_2O and MeOH molecule omitted for clarity together with all C hydrogen atoms. Magenta colour stands for low occupancy species associated with presence of low occupancy K^+ ions in the crown ether ring.

NMR experiments

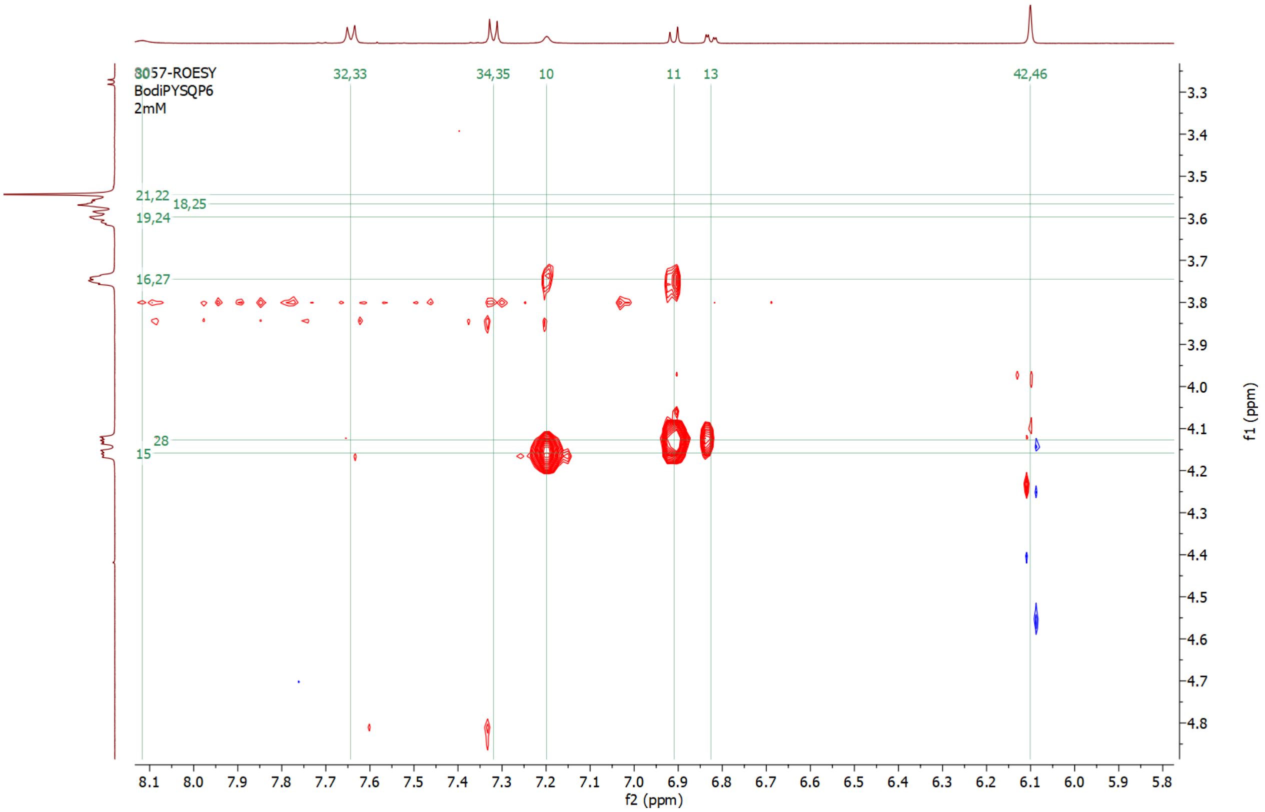

Figure S29. ROESY NMR spectrum of B1 (2 mM) in CD_3CN.

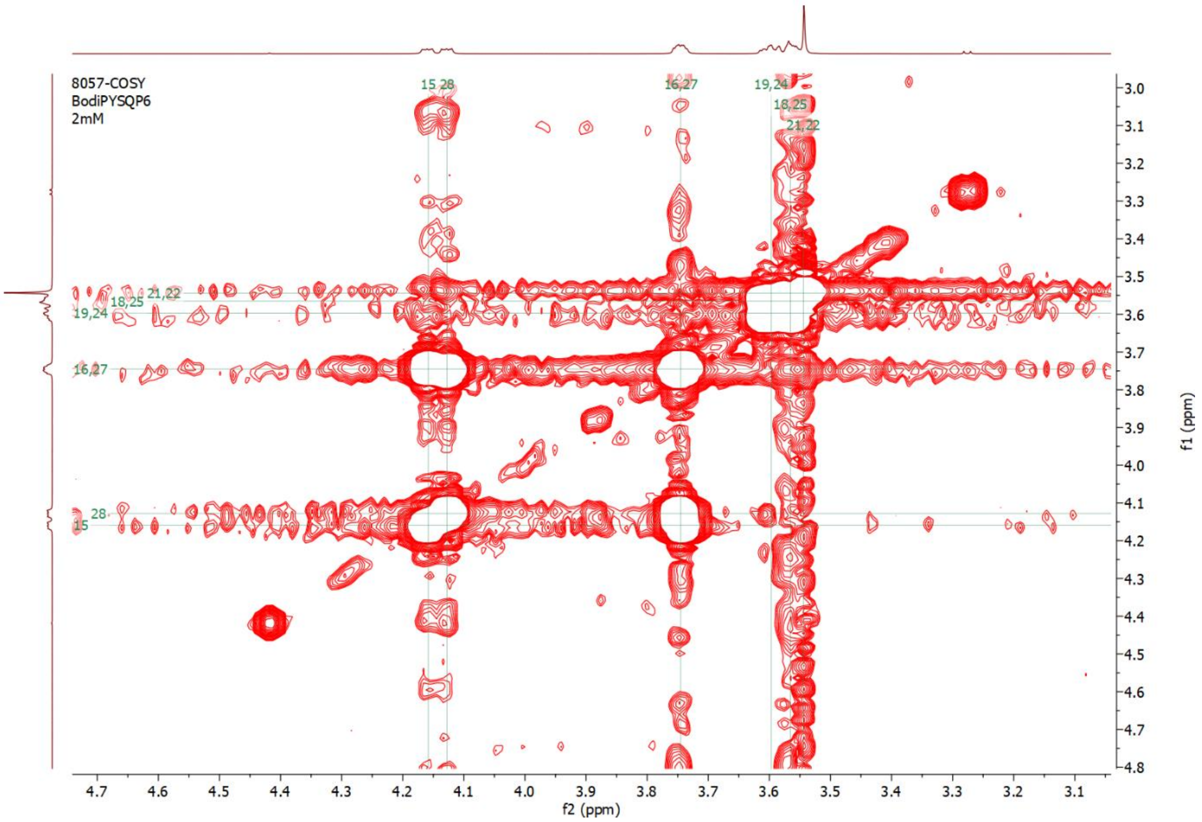

Figure S30. COSY NMR spectrum of B1 (2 mM) in CD_3CN.

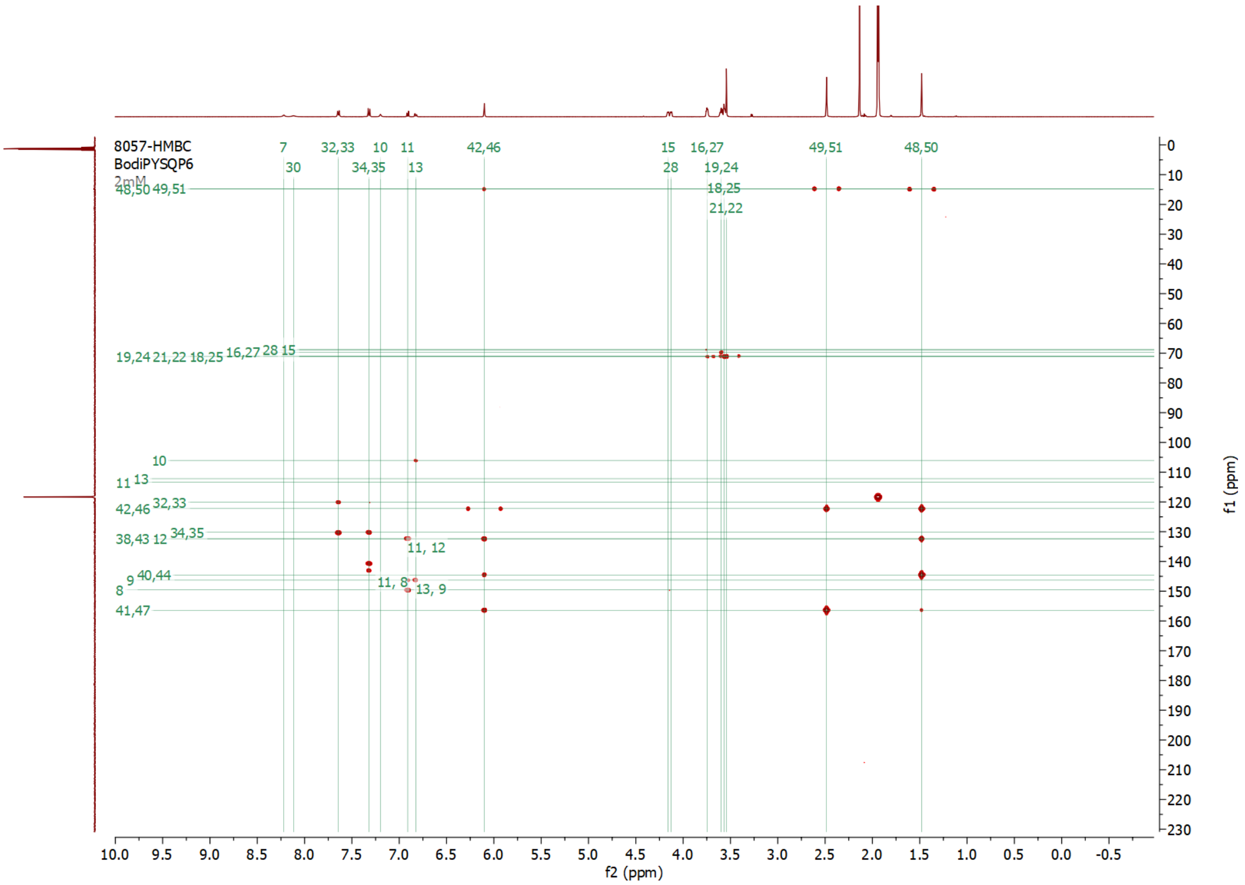

Figure S31. HMBC NMR spectrum of B1 (2 mM) in CD_3CN.

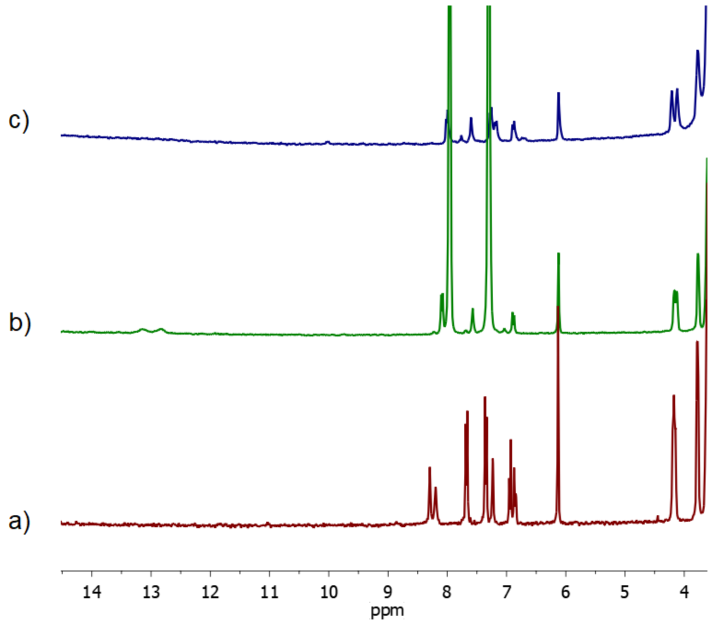

Figure S32. Partial ^{1}H NMR spectra (a) of receptor **B1** at 3 mM in CD_3CN; (b) upon addition >100 equiv. of TBAPhCOO; (c) upon addition >100 equiv. of $TBACH_3COO$.

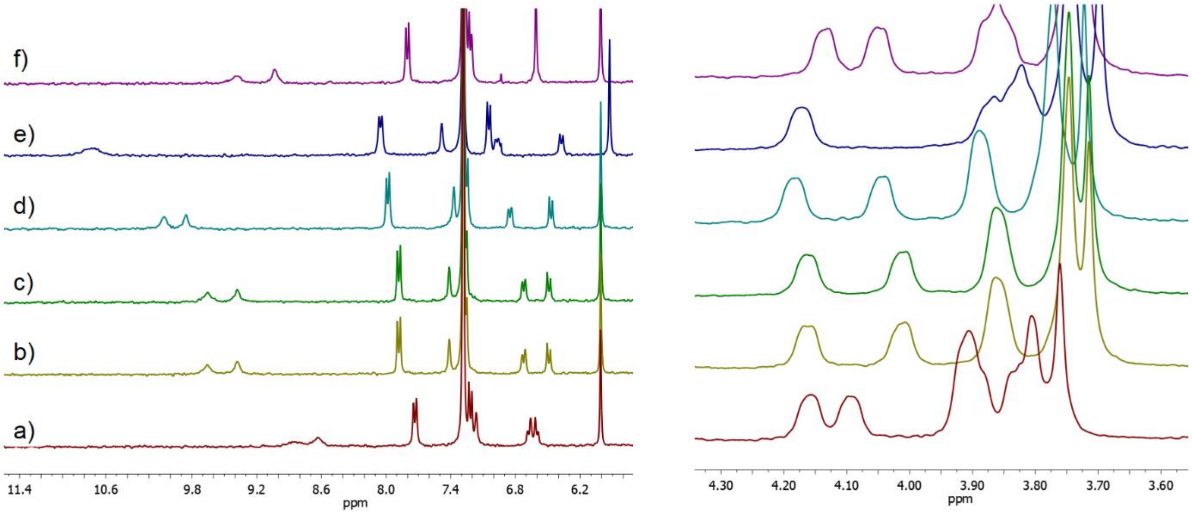

Figure S33. Partial ^{1}H NMR spectra of receptor **B1** at 3 mM (a) in wet $CDCl_3$; (b) after KNO_3 (20 mM) extraction from the aqueous phase; (c) after KBr (20 mM) extraction from the aqueous phase; (d) after KCl (20 mM) extraction from the aqueous phase; (e) after K_2SO_4 (20 mM) extraction from the aqueous phase; (f) after back extraction of the solution to distilled water.

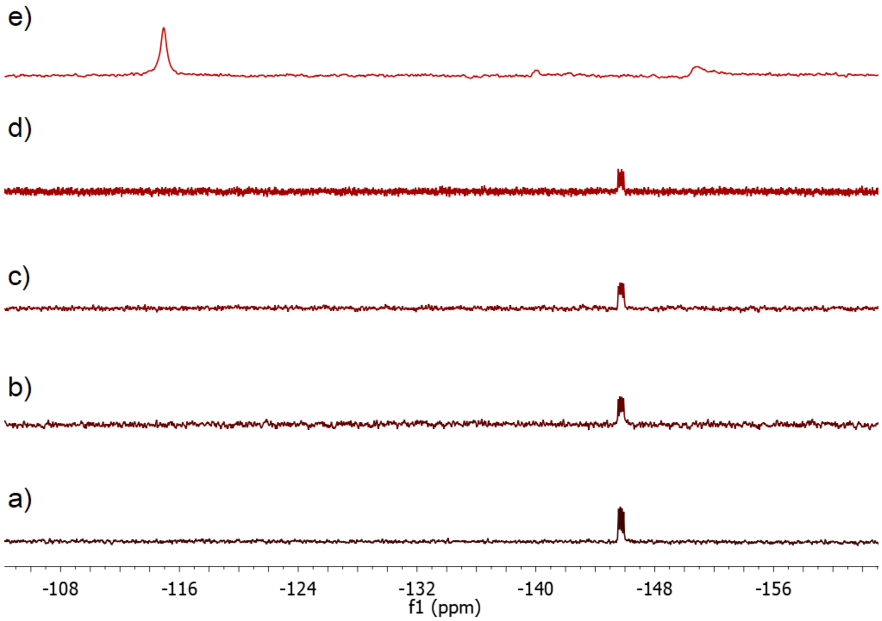

Figure S34. ^{19}F NMR spectra (a) of B3; after the addition of 50 equiv. of: (b) $TBACH_3COO$, (c) TBACl, (d) TBA_2SO_4, (e) TBAF in CD_3CN.

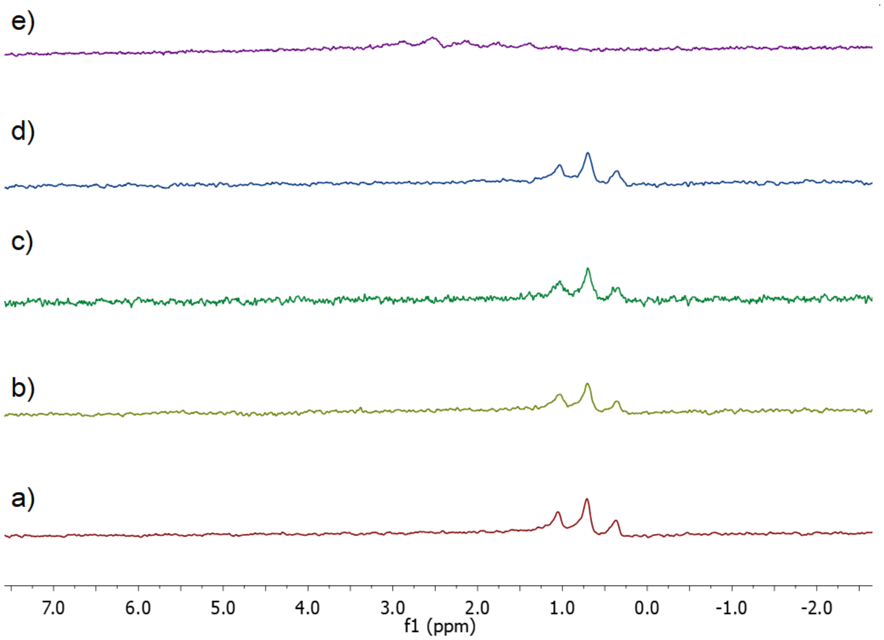

Figure S35. ^{11}B NMR spectra (a) of B3; after the addition of 50 equiv. of: (b) $TBACH_3COO$, (c) TBACl, (d) TBA_2SO_4, (e) TBAF in CD_3CN.

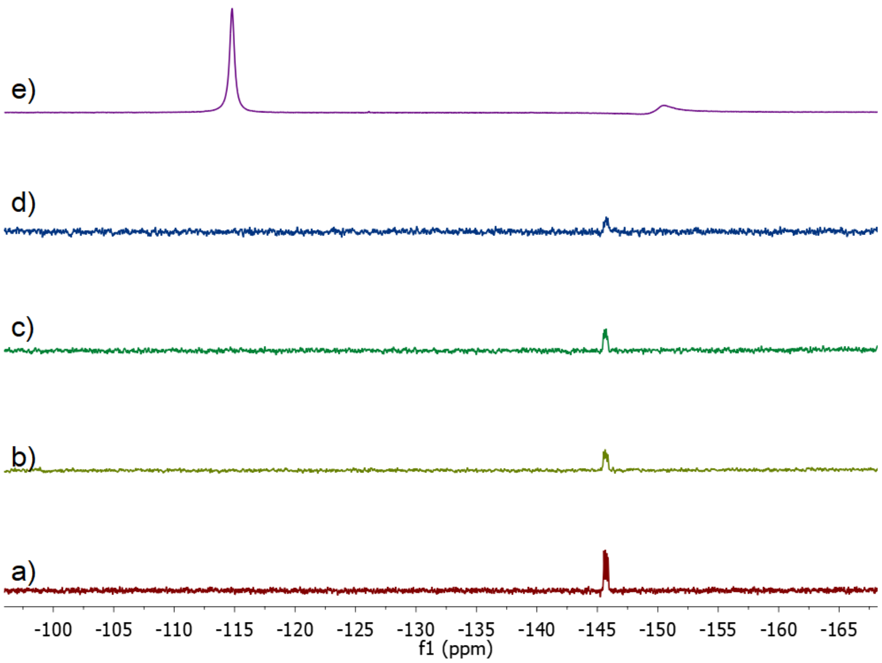

Figure S36. ^{19}F NMR spectra (a) of B1; after the addition of 50 equiv. of: (b) TBACl, (c) TBA_2SO_4, (d) $TBACH_3COO$, (e) TBAF in CD_3CN.

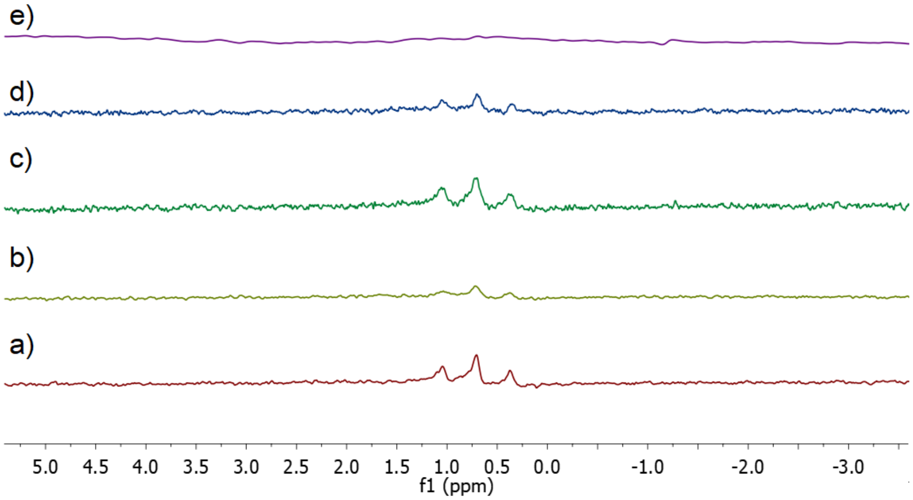

Figure S37. ^{11}B NMR spectra (a) of B1; after the addition of 50 equiv. of: (b) TBACl, (c) TBA_2SO_4, (d) $TBACH_3COO$, (e) TBAF in CD_3CN.

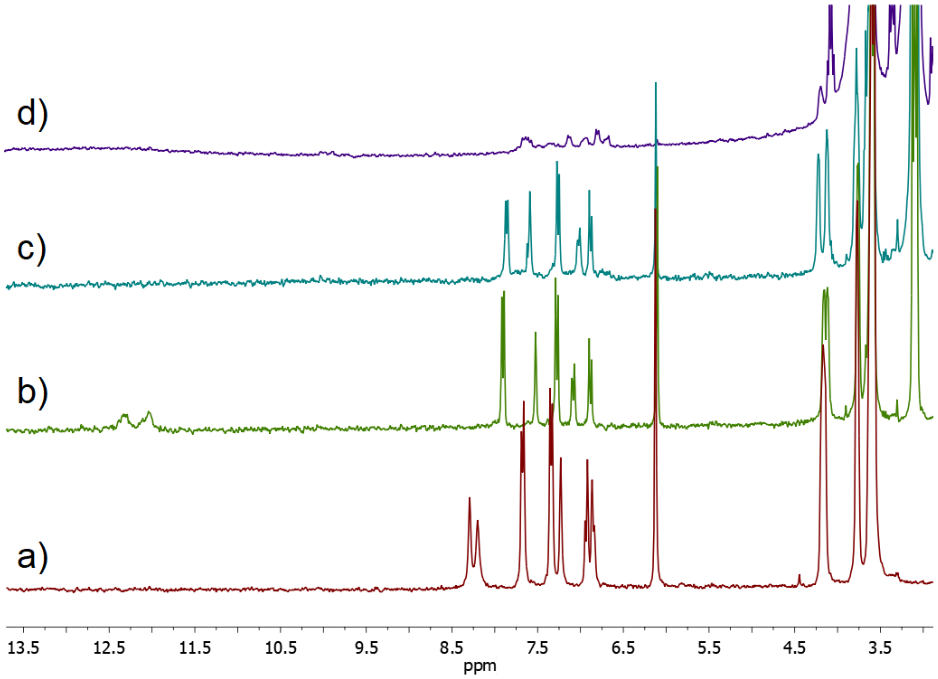

Figure S38. ^{1}H NMR spectra (a) of 3 mM B1 (ON1) (b) after the addition of 5 equiv. TBAF (OFF), (c) after the addition of 20 equiv. TBAF (ON2 deprotonation), (d) after the addition excess of TBAF (ON3) in CD_3CN.

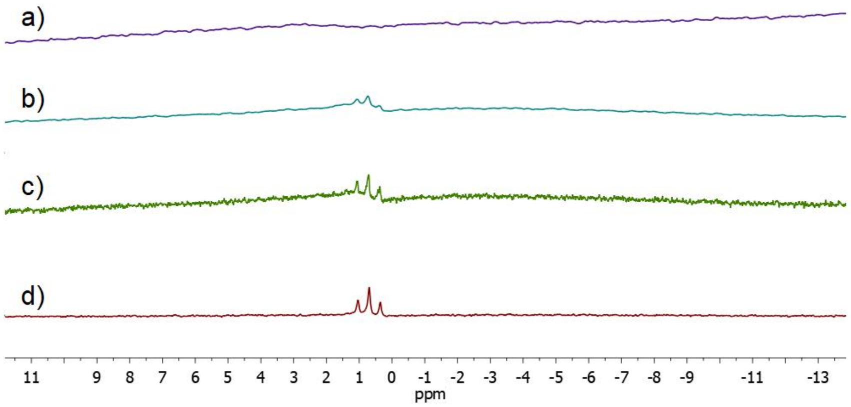

Figure S39. ^{11}B NMR spectra (a) of 3 mM B1 (ON1) (b) after the addition of 5 equiv. TBAF (OFF), (c) after the addition of 20 equiv. TBAF (ON2 deprotonation), (d) after the addition excess of TBAF (ON3) in CD_3CN.

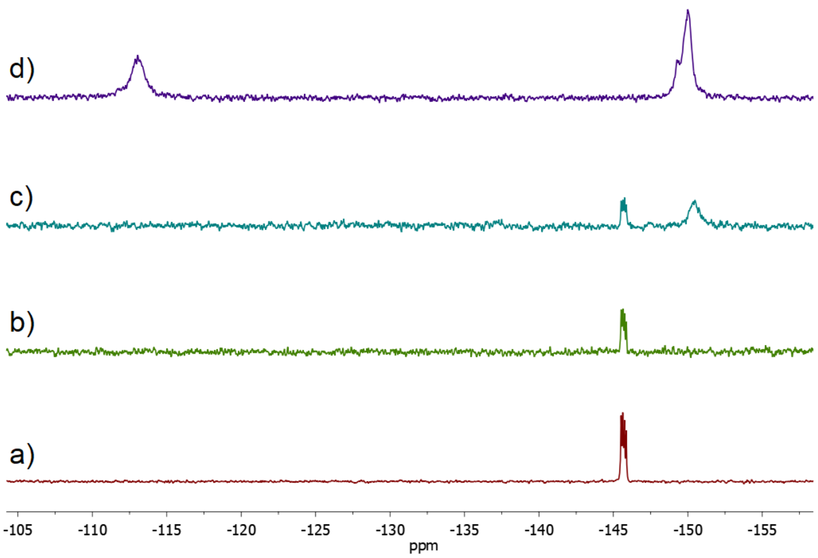

Figure S40. ^{19}F NMR spectra (a) of 3 mM B1 (ON1) (b) after the addition of 5 equiv. TBAF (OFF), (c) after the addition of 20 equiv. TBAF (ON2 deprotonation), (d) after the addition excess of TBAF (ON3) in CD_3CN.

Dynamic light scattering measurements (DLS)

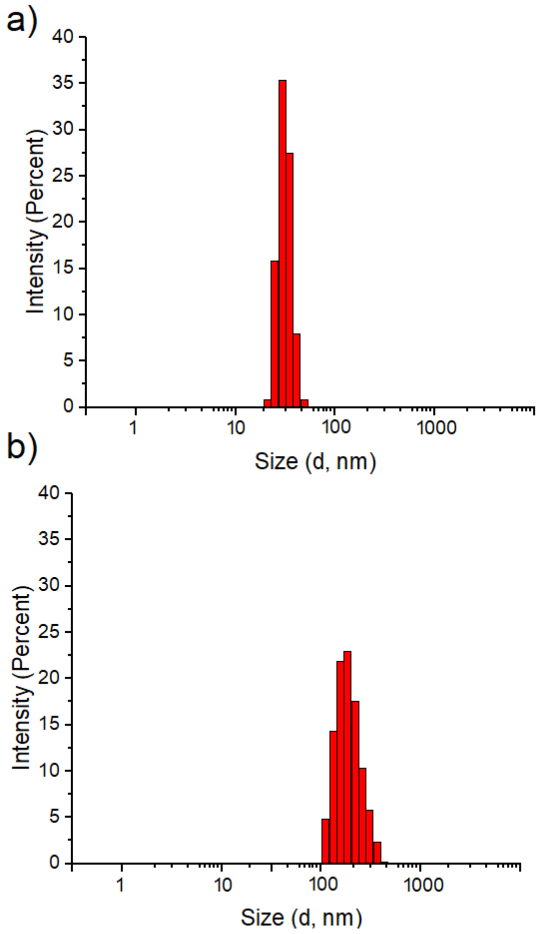

Figure S41. Distribution of the hydrodynamic diameter (a) of B1 (2.0×10^{-5}) in DMSO/ water (1:9); (b) B1 in DMSO/ 0.5 M aq. KCl.

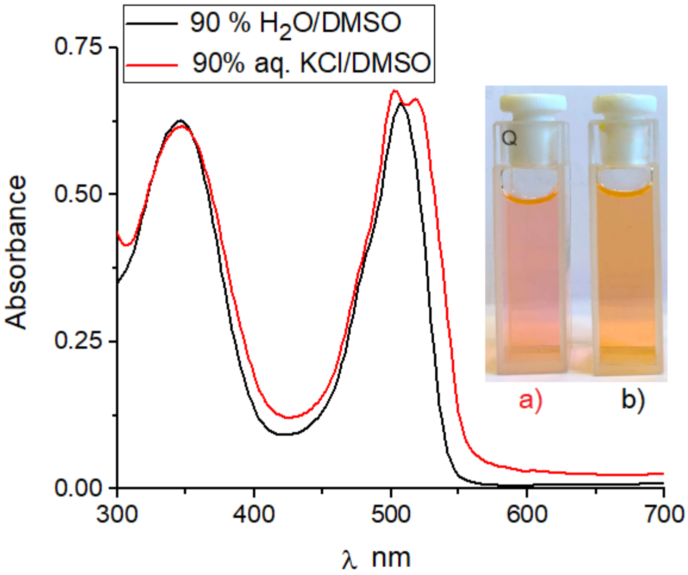

Figure S42. Absorption spectra (a) of B1 in DMSO/ 0.5 M aq. KCl (1:9) and (b) in DMSO/ water (1:9).

Extraction experiments - ion chromatography

A solution of receptor B1 in chloroform (2 ml, 20 mM) was intensive shaking with aqueous mixture (no pH adjustment, pH depending on the salts used; above pH 8 there is no phase separation probably due to the receptor deprotonation. This eliminates the direct use of basic salts such as carboxylates, hydrogen phosphates or phosphates. The receptor solution was shaken vigorously with the corresponding 5 mM saline solution (2 ml) for 5 minutes. Then 1 mL of the aqueous phase was taken and tenfold diluted. The concentration of chloride, bromide, nitrite, nitrate, dihydrogenphosphate and sulfate anions in the aqueous phase was determined by high performance ion chromatography (HPIC).

Table S1. Summarized data for competitive LLE. Source phase: aqueous of potassium salts (5 mM each); Organic phase: B1 (20 mM) in $CHCl_3$.

Anion	Source phase [mg/l]	After extraction with B1 [mg/l]	Decrease in salt concentration after extraction [%]
Cl^-	17.75	13.12	26
Br^-	40.00	26.42	34
NO_3^-	31.00	22.57	27
SO_4^{2-}	48.00	34.20	29

Transport Studies

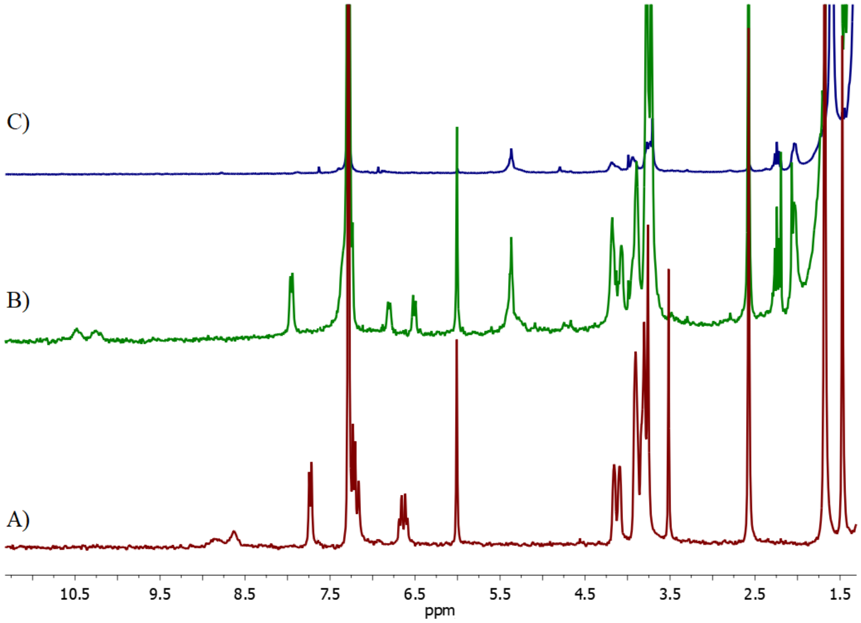

Figure S43. Partial ^{1}H NMR spectra A) of receptor B1 recorded in aq. $CDCl_3$; B) Receiving phase after conducting the B1 transport experiment by the saturated KCl, after 7 days; C) Receiving phase after conducting the B1 transport experiment by water, after 7 days.

Detection limit

The detection limit was determined from the fluorescence titration data based on a reported method.[S8-S10] The fluorescence spectrum of B1 was measured by twenty times and the standard deviation of blank measurement was achieved. To gain the slop, the fluorescent intensity data at 526 nm was plotted as a concentration of anion (Cl^- or Br^-). So the detection limit was calculated with the following equation:

$$Detection\ limit = 3\sigma/k$$

Where σ is the standard deviation of blank measurement, and k is the slop between the fluorescence versus anion concentration. There was a good linearity at micro molar concentration levels between fluorescent intensity data at 526 nm and concentrations of anion in the range from 4×10^{-6} M to 18×10^{-6} M, indicating the probe B1 can detect quantitatively relevant concentrations of anion.

The linear equation was found to be $y=-(7.4978x-1119.5)$($R^2=0.955$), where y is the fluorescent intensity data at 526 nm measured at a given Cl^- concentration and x represents the concentration of Cl^- added. So the detection limit for Cl^- was calculated to be 1.2×10^{-7} (Detection limit$=3\sigma/k=0.88/7.4978\times10^{-6}$ M $\approx 1.2 \times 10^{-7}$).

The linear equation was found to be $y=-(2.2075x-1118.5)$($R^2=0.955$), where y is the fluorescent intensity data at 526 nm measured at a given Br^- concentration and x represents the concentration of Br^- added. So the detection limit for Br^- was calculated to be 4×10^{-7} (Detection limit$=3\sigma/k=0.88/2.2075\times10^{-6}$ M $\approx 4 \times 10^{-7}$).

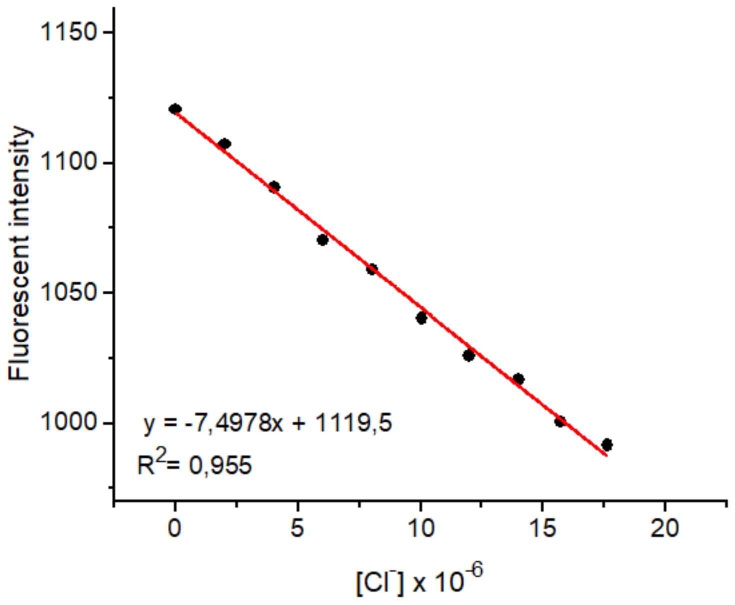

Figure S44. Response of fluorescence signal of B1 in CH_3CN (2.0×10^{-5} M) in the presence of increasing amount of Cl^- (0 M to 18×10^{-6} M) (excitation at 496 nm).

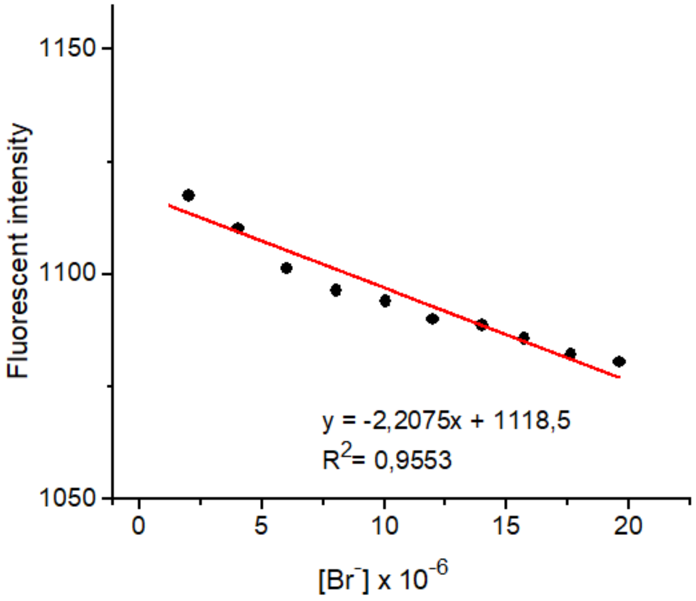

Figure S45. Response of fluorescence signal of B1 in CH_3CN (2.0×10^{-5} M) in the presence of increasing amount of Br^- (4 M to 20×10^{-6} M) (excitation at 496 nm).

Fluorescent experiments

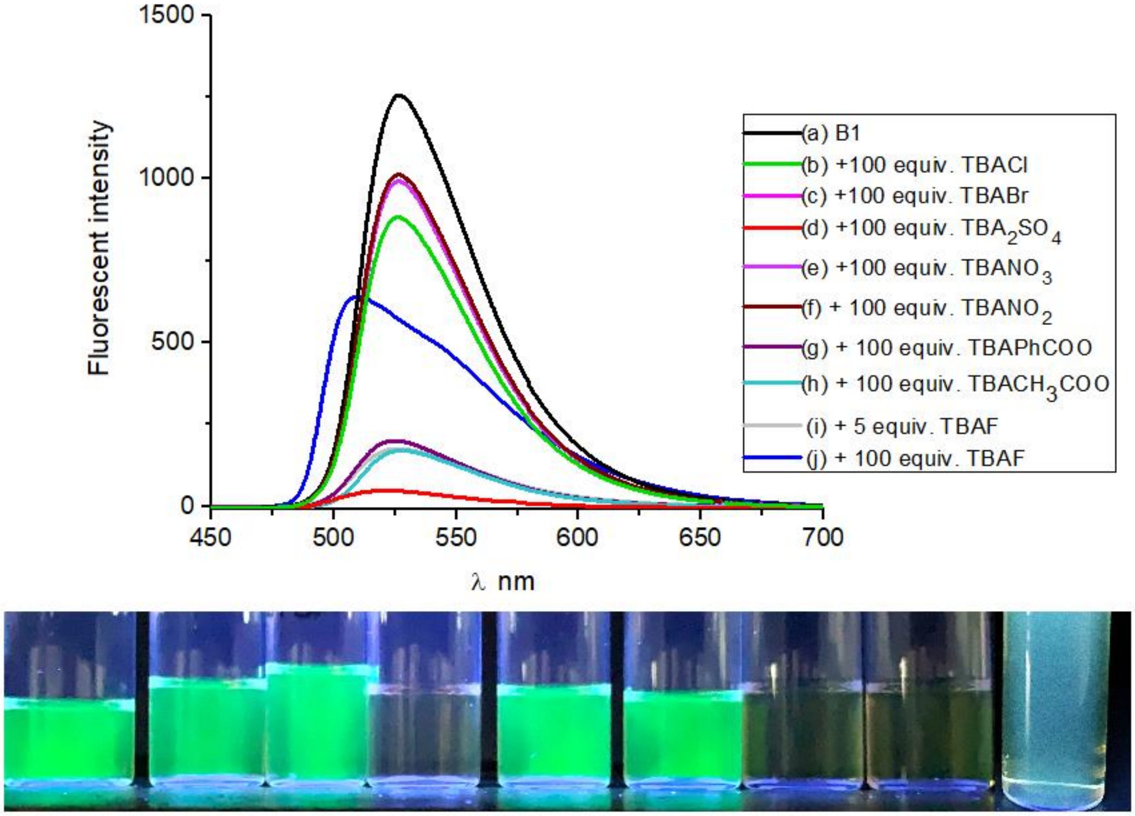

a b c d e f g h j

Figure S46. Emission spectra of B1 (2.0×10^{-5} M) and upon addition of 100 equiv. TBA salts (excitation at 496 nm) in CH_3CN.

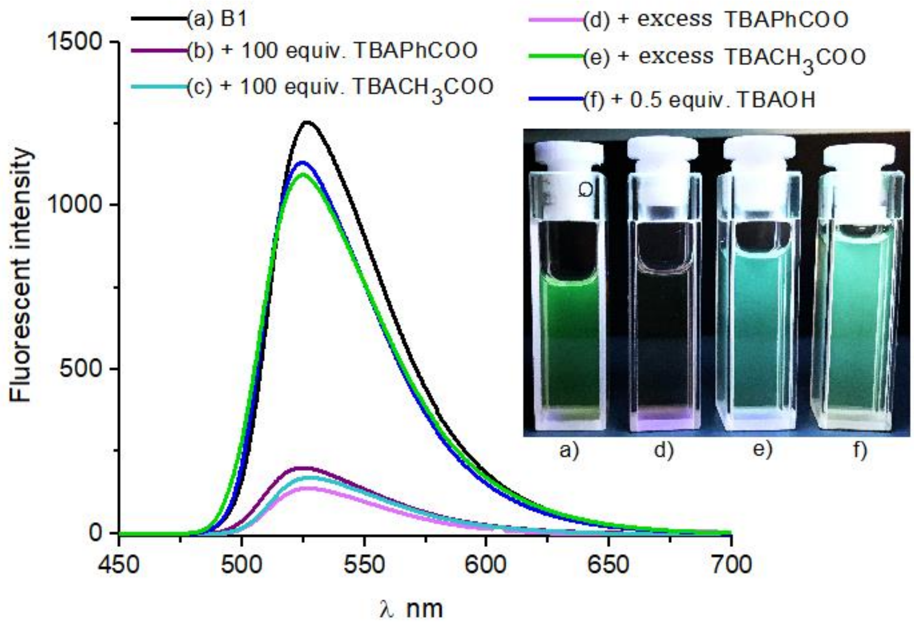

Figure S47. Emission spectra of B1 (2.0 × 10^{-5} M) and upon addition of TBA salts (excitation at 496 nm) in CH_3CN.

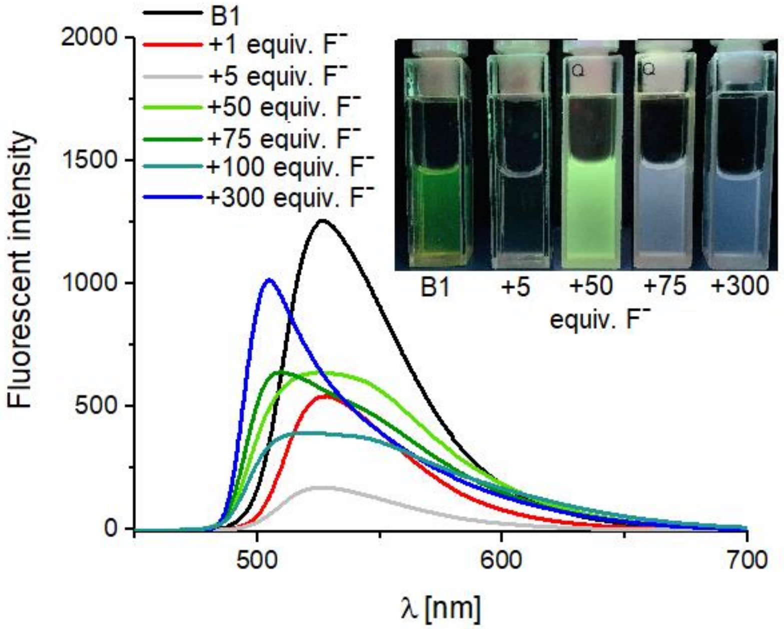

Figure S48. Emission spectra of B1 (2.0 × 10^{-5} M) and upon addition of TBAF in CH_3CN.

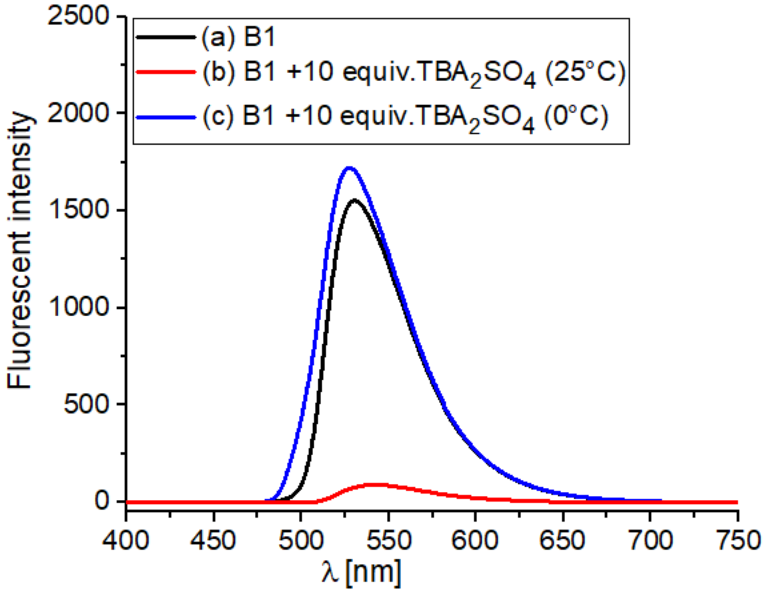

Figure S49. Emission spectra a) of Receptor B1 (2.0×10^{-5} M); b) upon addition 10 equiv. of TBA_2SO_4 at 25°C and c) at 0°C (excitation at 496 nm) in CH_3CN.

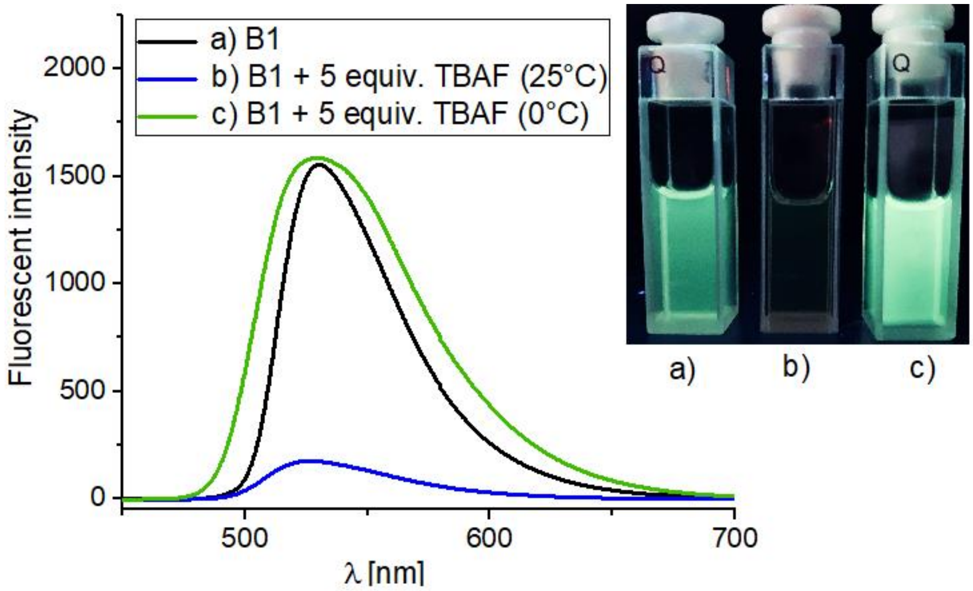

Figure S50. Emission spectra a) of Receptor B1 (2.0×10^{-5} M), b) upon addition 10 equiv. of TBAF at 25°C and c) at 0°C (excitation at 496 nm) in CH_3CN.

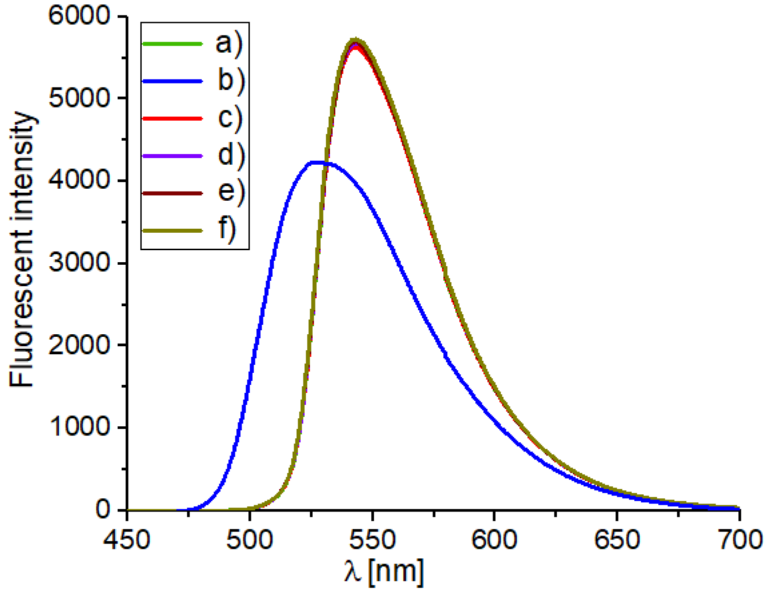

Figure S51. Emission spectra a) of B3 (2.0×10^{-5} M) and b) upon addition 100 equiv. of TBAF; c) upon addition 100 equiv. of TBACl; d) upon addition 100 equiv. of TBABr; e) upon addition 100 equiv. of TBA_2SO_4; f) upon addition 100 equiv. of $TBACH_3COO$ (excitation at 496 nm) in CH_3CN.

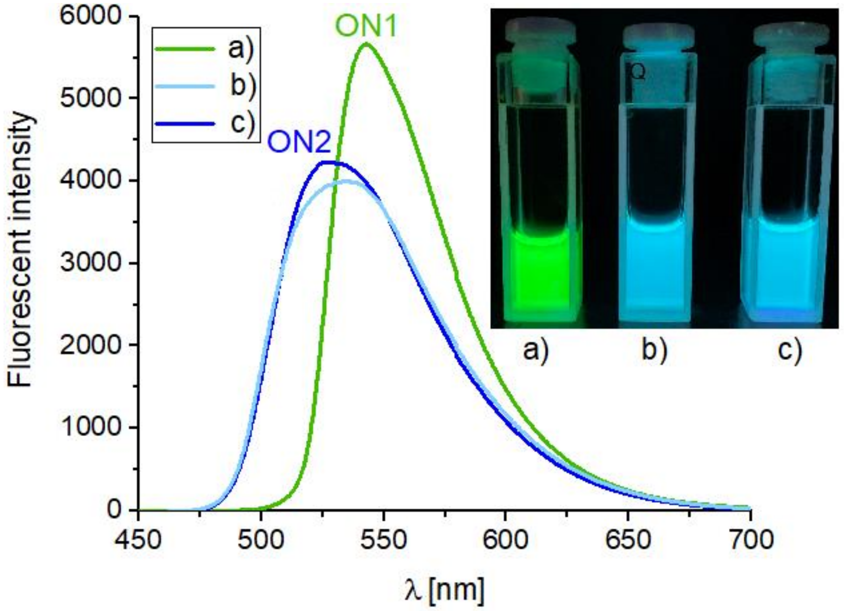

Figure S52. Emission spectra a) of B3 (2.0×10^{-5} M) and b) upon addition 5 equiv. of TBAF; c) upon addition 100 equiv. of TBAF.

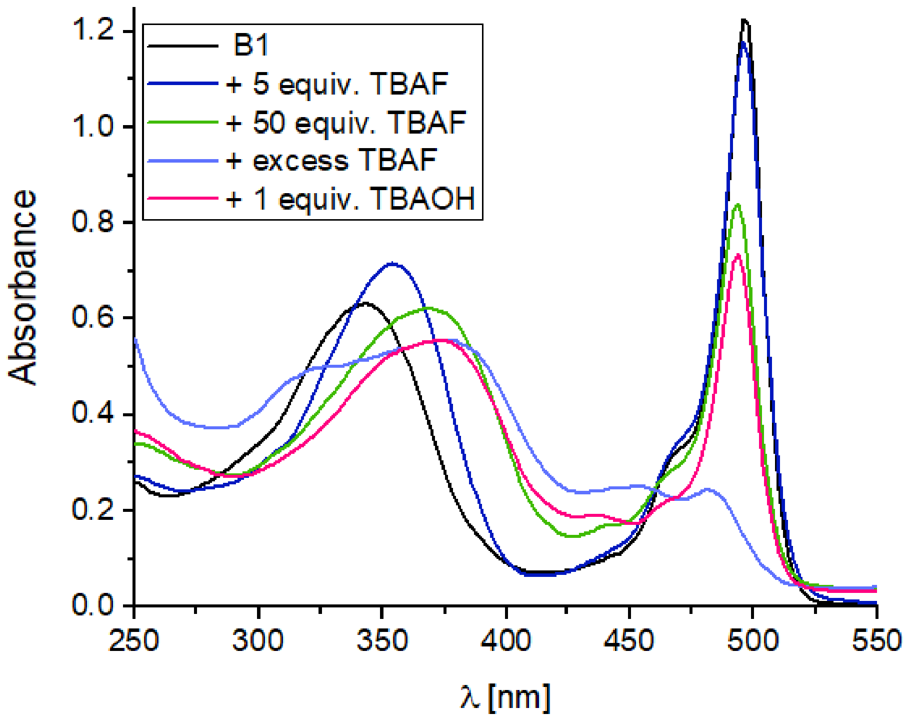

Figure S53. Absorption spectra of B1 (2.0 ×10^{-5} M) in CH_3CN and after addition of TBAF or TBAOH.

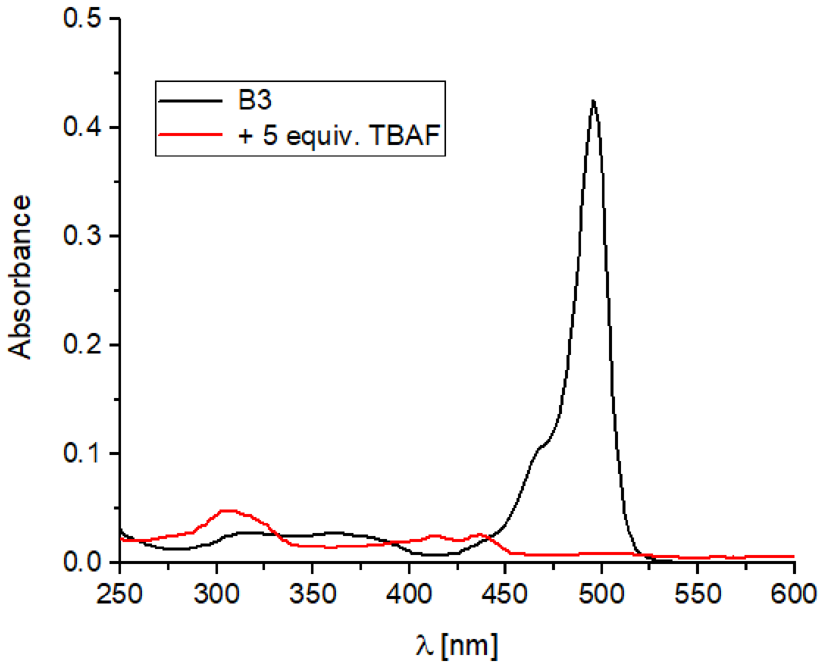

Figure S54. Absorption spectra of B3 (2.0 ×10^{-5} M) in CH_3CN and after addition of TBAF.

References

S1. APEX2; Bruker AXS Inc.: Madison, WI, USA, 2017.

S2. SAINT; Bruker AXS Inc.: Madison, WI, USA, 2017.

S3. SADABS; Bruker AXS Inc.: Madison, WI, USA, 2016.

S4. Sheldrick, G.M. SHELXT-Integrated Space-Group and Crystal-Structure Determination. Acta Cryst. 2015, A71, 3–8.

S5. Sheldrick, G.M. Crystal Structure Refinement with SHELXL. Acta Cryst. 2015, C71, 3–8.

S6. International Tables for Crystallography; Wilson, A.J.C., Ed.; Kluwer: Dordrecht, The Netherlands, 1992.

S7. Macrae, C.F.; Sovago, I.; Cottrell, S.J.; Galek, P.T.A.; McCabe, P.; Pidcock, E.; Platings, M.; Shields, G.P.; Stevens, J.S.; Towler, M.; et al. Mercury 4.0: From visualization to analysis, design and prediction. J. Appl. Crystallogr. 2020, 53, 226–235.

S8. Ma, Q.J.; Zhang, X.B.; Zhao, X.H.; Jin, Z.; Mao, G.J.; Shen, G.L.; Yu, R.Q. A highly selective fluorescent probe for Hg2+ based on a rhodamine–coumarin conjugate. Anal. Chim. Acta 2010, 663, 85.

S9. Li, Y.M.; Zhang, X.L.; Zhu, B.C.; Yan, J.L.; Xu, W.P. A Highly Selective Colorimetric and “Off-on-off” Fluorescent Probe for Fluoride Ions. Anal. Sci. 2010, 26, 1077.

S10. Yang, X.F.; Wang, L.P.; Xu, H.M.; Zhao, M.L. Anal. Chim. Acta 2009, 631, 91.
